# Design, Synthesis, and Biological Evaluation of Novel Quinazolin-4(3H)-One-Based Histone Deacetylase 6 (HDAC6) Inhibitors for Anticancer Activity

**DOI:** 10.3390/ijms241311044

**Published:** 2023-07-03

**Authors:** Yogesh Mahadu Khetmalis, Ashna Fathima, Markus Schweipert, Cécile Debarnot, Naga Venkata Madhusudhan Rao Bandaru, Sankaranarayanan Murugesan, Trinath Jamma, Franz-Josef Meyer-Almes, Kondapalli Venkata Gowri Chandra Sekhar

**Affiliations:** 1 Department of Chemistry, Birla Institute of Technology & Science-Pilani, Hyderabad Campus, Hyderabad 500078, Telangana, India; yogeshkhetmalis88@gmail.com (Y.M.K.); madhusudhan_bandaru123@yahoo.co.in (N.V.M.R.B.); 2 Department of Biological Sciences, Birla Institute of Technology & Science-Pilani, Hyderabad Campus, Hyderabad 500078, Telangana, India; p20210001@hyderabad.bits-pilani.ac.in (A.F.); trinath@hyderabad.bits-pilani.ac.in (T.J.); 3 Department of Chemical Engineering and Biotechnology, University of Applied Sciences Darmstadt, Haardtring 100, 64295 Darmstadt, Germany; markus.schweipert@h-da.de (M.S.); 2210589@stud.hs-mannheim.de (C.D.); 4 Medicinal Chemistry Research Laboratory, Department of Pharmacy, Birla Institute of Technology & Science Pilani, Pilani Campus, Pilani 333031, Rajasthan, India; murugesan@pilani.bits-pilani.ac

**Keywords:** cancer, histone deacetylase, quinazoline-4-(3H)-one, small-molecule inhibitors

## Abstract

A series of novel quinazoline-4-(3H)-one derivatives were designed and synthesized as histone deacetylase 6 (HDAC6) inhibitors based on novel quinazoline-4-(3H)-one as the cap group and benzhydroxamic acid as the linker and metal-binding group. A total of 19 novel quinazoline-4-(3H)-one analogues (**5a**–**5s**) were obtained. The structures of the target compounds were characterized using ^1^H-NMR, ^13^C-NMR, LC–MS, and elemental analyses. Characterized compounds were screened for inhibition against HDAC8 class I, HDAC4 class IIa, and HDAC6 class IIb. Among the compounds tested, **5b** proved to be the most potent and selective inhibitor of HDAC6 with an IC_50_ value 150 nM. Some of these compounds showed potent antiproliferative activity in several tumor cell lines (HCT116, MCF7, and B16). Amongst all the compounds tested for their anticancer effect against cancer cell lines, **5c** emerged to be most active against the MCF-7 line with an IC_50_ of 13.7 μM; it exhibited cell-cycle arrest in the G2 phase, as well as promoted apoptosis. Additionally, we noted a significant reduction in the colony-forming capability of cancer cells in the presence of **5c**. At the intracellular level, selective inhibition of HDAC6 was enumerated by monitoring the acetylation of α-tubulin with a limited effect on acetyl-H3. Importantly, the obtained results suggested a potent effect of **5c** at sub-micromolar concentrations as compared to the other molecules as HDAC6 inhibitors in vitro.

## 1. Introduction

Cancer is the second leading cause of death worldwide. In 2020, 10 million cancer deaths were reported by the World Health Organization (WHO). Due to the rising prevalence of cancer, much effort is put toward designing and synthesizing possible anticancer medicines in medicinal chemistry [1,2,3,4,5]. Histone acetyl transferases (HATs) and histone deacetylases (HDACs) are the enzymes that control the process of intracellular histone acetylation. HATs function as “writers” transferring the acetyl group from acetyl-CoA to the amine group of lysine residues on histones and non-histone proteins, expanding the chromosome’s landscape to be detected by appropriate transcription factors or regulatory proteins, later recruiting transcription factors for subsequent gene expression [6,7,8,9,10,11]. As a result, significant research priority is given to the development of isozyme-selective HDAC inhibitors with fewer adverse effects and increased potency. However, chemotherapies must first and foremost be effective, preferably as a single agent, and toxicity must be acceptable compared to efficacy. Histone deacetylase 6 (HDAC6) is a class II enzyme that belongs to the class IIb category. It differs structurally and functionally from other HDACs and is predominantly found in the cytoplasm. HDAC6 is a 1255-amino-acid protein that is one of the largest in the HDAC family. HDAC6 is associated with immune synapse, microtubule-dependent cell motility, and viral infection [12,13]. 

HDAC6 plays significant roles in multiple processes associated with tumorigenesis, including increased cellular proliferation, migration, and invasion of cancer cells [14,15,16]. Interestingly, HDAC6 inhibitors also show immunomodulatory properties with potential to be used as therapeutic agents for cancer immunotherapy [17]. The inhibition of HDAC6 in melanoma tumor cells resulted in a reduction in the expression of the immunosuppressive molecule PD-L1. This effect was achieved by influencing the recruitment and activation of STAT3 [18]. Since HDAC6 is structurally and functionally distinctly different from the other HDAC isozymes, selectively targeting HDAC6 is supposed to maximize the pharmacological effects, while minimizing the side-effects associated with pan-HDAC inhibitors [19,20]. This hypothesis also stimulated this study to develop new HDAC6-selective inhibitors. Cheng et al. used quinazoline as the cap group and benzhydroxamic acid moiety as the linker and zinc-binding group to develop HDAC6 inhibitors. The best compound from this work exhibited an IC_50_ of 4000 nM [21]. In our current work, with a slight variation in the substituents, using quinazoline as the cap group and benzhydroxamic acid moiety as the linker and zinc-binding group, we obtained HDAC6 inhibitors with improved potency. Several compounds exhibited an IC_50_ < 900 nM while the most active compound showed an IC_50_ of 150 nM. 

**Figure 1:**
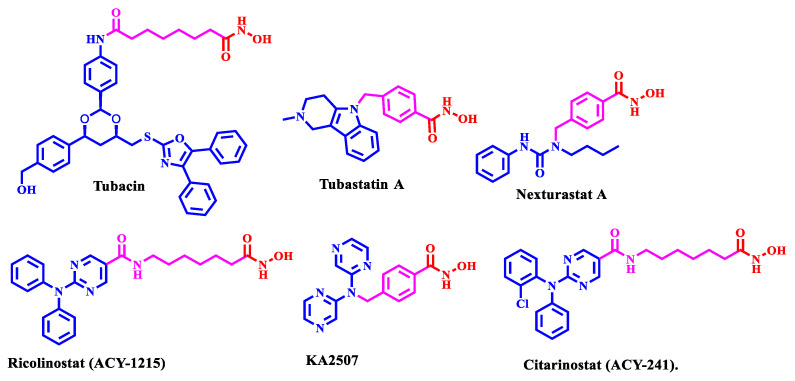
Structures of some selective HDAC6 inhibitors and those currently in clinical trials.

Figure 1 shows the structures of some selective HDAC 6 inhibitors and those that are currently in clinical trials [22,23,24,25]. 

Ricolinostat and KA2507 have already been investigated in phase I/II clinical trials. KA2507 showed selective target engagement, no significant toxicities, and prolonged dis-ease stabilization in a subset of patients [25]. Ricolinostat was tested as a single agent, in combination with paclitaxel in patients with metastatic breast cancer, and in combination with bortezomib and dexamethasone in multiple myeloma therapy. Single-agent therapy showed neither significant toxicities nor clinical responses. Ricolinostat could be safely combined with paclitaxel; clinical activity was identified in a particular patient group, and the HDAC6 score was shown to have potential as a predictive biomarker [26]. Combination therapy with bortezomib and dexamethasone was shown to be safe, well-tolerated, and active against multiple myeloma [27]. These results are promising, but still leave much room for HDAC6 inhibitors with fewer side-effects but more efficacy.

### The Basic Structure of Zinc-Binding HDAC Inhibitors

**Figure 2:**
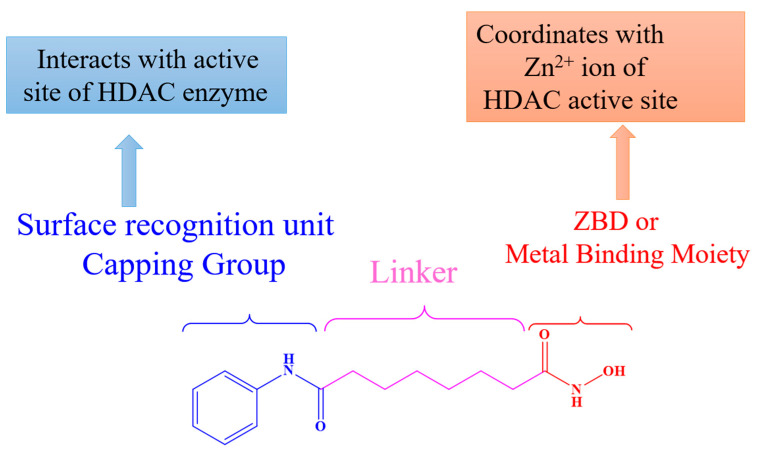
General pharmacophore model of HDAC inhibitors.

The general pharmacophore model of HDAC inhibitors acting through the zinc-binding mechanism is represented in Figure 2. This pharmacophore model consists of three basic units: (i) cap group, (ii) linker function, and (iii) Zn-binding group [28,29,30,31,32].

## 2. Results and Discussion

**Figure 3:**
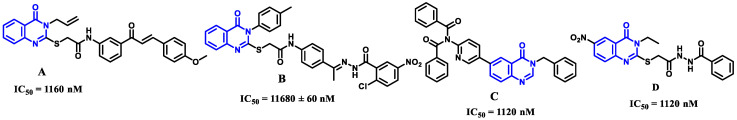
Structures of quinazoline-based anticancer compounds.

Quinazolines are privileged scaffolds that are used as therapeutic targets in medicinal chemistry with versatile pharmacophoric properties. They exhibit anticancer, analgesic, anti-inflammatory, antibacterial, anticonvulsant, antidiabetic, antihypertensive, and dihydrofolate reductase-inhibitory effects [33,34,35,36,37,38]. Quinazolines were recently reported as anticancer agents by various groups. Mohamed Hisham and colleagues developed quinazoline-4-one derivatives as EGFR inhibitors for anticancer activity. With an IC_50_ value of 1160 nM, compound **A** was the most active in all cell lines examined (A549, MCF-7, and HT-29) [39]. Khaled El-Adl et al. reported quinazolin-4(3H)-ones as potential inhibitors of VEGFR-2. The most active component was compound **B**, which had a 2-chloro-5-nitrophenyl group. It was more effective against HepG2, HCT-116, and MCF-7 cells than doxorubicin against the HCT-116 cell line, with an IC_50_ of 11,680 ± 60 nM [40]. Novel quinazolin-4(3H)-one compounds were described by Huarong Yang and colleagues as possible anticancer drugs. Several derivatives showed antiproliferative effect against the cancer cells that were studied. Compound **C**, with an IC_50_ value of 1120 nM, was the strongest inhibitory agent against all cancer cell lines and was even more powerful against HCC827 cells [41]. Abdallah E. Abdallah reported the synthesis and biological evaluation of novel quinazolin-4(3H)-one based VEGFR-2 kinase inhibitors for anticancer activity. Several compounds showed antiproliferative effects against the cancer cells that were examined. Compound **D**, with an IC_50_ value of 1120 nM, exhibited the strongest inhibitory effect against all cancer cell lines and was even more powerful against HCC827 cells (Figure 3) [42]. 

**Figure 4:**
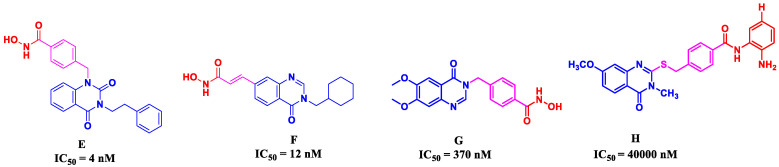
Structures of HDAC inhibitors comprising quinazoline as the cap group.

Numerous studies have identified quinazoline-based hydroxamic acids as hybridized deacetylase inhibitors and anticancer agents [43]. Hydroxamic acids based on quinazolin-2,4-dione were developed and synthesized by the Chao-Wu Yu group to act as selective competitive inhibitors of HDAC6. Among the HDAC6 inhibitors reported by this group, the most effective and selective compound was **E** with an IC_50_ value of 4 nM [44]. Duong et al. reported novel 4-oxoquinazoline-based compounds and tested them for HDAC inhibition and cytotoxicity effects against three human cancer cell lines. Most of these compounds displayed good to potent HDAC inhibitory efficacy and cytotoxicity (SW620—colon cancer, PC-3—prostate cancer, and NCI-H23—lung cancer). With an IC_50_ value of 12 nM, compound **F** was the most potent HDAC6 inhibitor [45]. Doan Thanh Hieu and colleagues developed novel small compounds for HDAC inhibition. They designed and synthesized new compounds with the quinazoline-4-(3H)-one as the cap group. In this study, compound **G** exhibited more potent cytotoxicity and HDAC inhibitory activity (IC_50_ = 370 nM) [43]. The Chunhui Cheng group developed and synthesized novel 2-aminobenzamide derivatives for HDAC inhibition by adding quinazolinone as the cap group. Compound **H** was the most potent against HDAC6 with an IC_50_ value of 40,000 nM. Furthermore, these compounds showed good antiproliferative action against various human cancer cell lines (Figure 4) [21]. 

**Figure 5:**
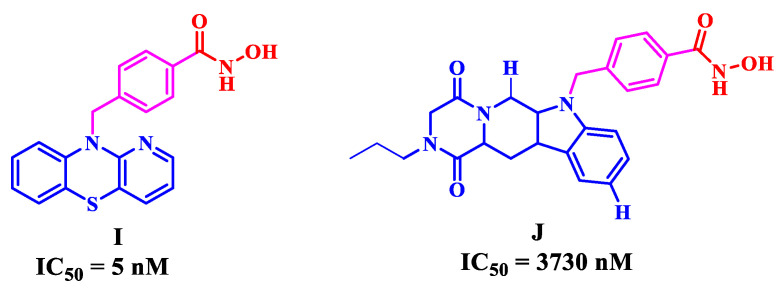
Structures of HDAC inhibitors comprising the benzhydroxamic acid moiety as the linker and zinc-binding group.

Katharina and coworkers reported a series of compounds with benzhydroxamic acid moiety as the linker and zinc-binding group. They evaluated these compounds for HDAC6 inhibition. In this series, compound **I** with an IC_50_ value of 5 nM emerged as the most potent and selective compound toward HDAC6 inhibition [46]. Michel Leonhardt’s group designed and synthesized a series of novel derivatives using benzhydroxamic acid moiety as the zinc-binding group for HDAC inhibition. They found that, among all, compound **J** was the most active with an IC_50_ value of 3730 nM for HDAC6 inhibition (Figure 5) [47].

### 2.1. Designing of the Molecules

**Figure 6:**
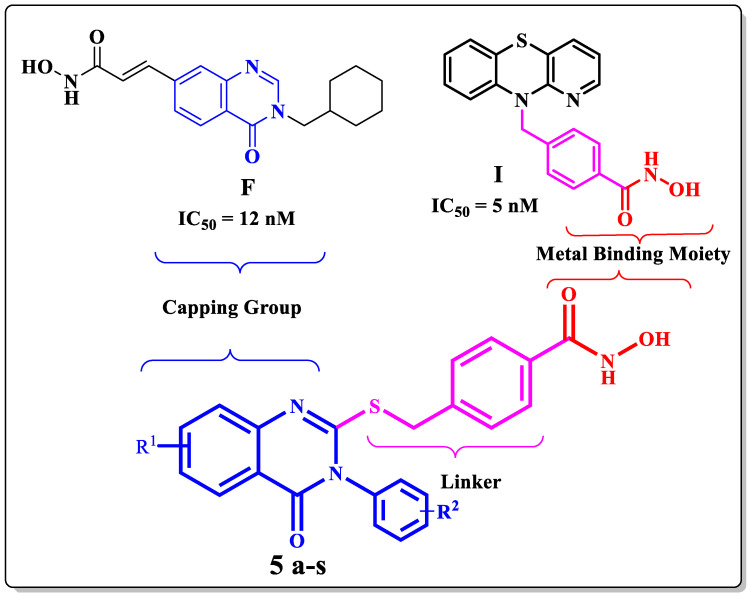
Scaffold design strategy for the current novel HDAC 6 inhibitors.

Although only a few HDAC inhibitors have been approved by the US Food and Drug Administration (FDA) for cancer treatment, most of these inhibitors act on several HDACs, are nonselective, and induce substantial side-effects, limiting their clinical application in oncology and beyond [48]. The approved HDAC inhibitors are effective as single agents and have only relatively tolerable side-effects. On the other hand, it is possible to reduce unwanted side-effects by the development of inhibitors for specific isozymes. As selective inhibition of HDAC6 has fewer adverse effects, we designed the compounds **5a**–**5s** (Figure 6) with a modification of the (quinazoline) cap region using distinct aromatic functionalities and the benzhydroxamic acid moiety as the linker and zinc-binding group to improve HDAC6 selectivity.

### 2.2. Chemistry

**Scheme 1:**
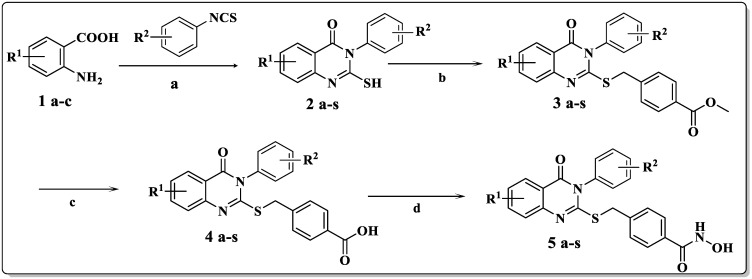
Reagents and conditions: (**a**) Et_3_N, EtOH, reflux, 12 h; (**b**) methyl 4-(bromomethyl) benzoate, KI, K_2_CO_3,_ butanone, 75 °C, 4 h; (**c**) NaOH, 1,4-dioxane, 80 °C, 4 h; (**d**) HBTU, DIPEA, NH_2_OH.HCl., DMF, rt, 10–16 h.

The synthesis of hydroxamic acid derivatives (**5a**–**5s**) having a quinazoline-4-(3H)-one (cap group) and benzhydroxamic acid as (linker and metal-binding group) was carried out as illustrated in Scheme 1. 

The synthesis of target molecules (**5a**–**5s**) was carried out using commercially available substituted anthranilic acid (**1a**–**c**) (Scheme 1) and different substituted phenyl isothiocyanates as the starting materials. Substituted anthranilic acids (**1a**–**c**) on refluxing in ethanol with substituted phenyl isothiocyanate yielded key intermediates (**2a**–**2s**) (Scheme 1). Compounds **3a**–**3s** were prepared by coupling intermediates **2a**–**2s** with 4-(bromomethyl)benzoate via an S-alkylation reaction. Hydrolysis of the esters (**3a**–**3s**) in the presence of NaOH and 1,4-dioxane gave the corresponding acids **4a**–**4s**. These intermediates were directly coupled with hydroxylamine hydrochloride via an amide coupling reaction to obtain the desired target compounds (**5a**–**5s**) (Scheme 1). The structures of the designed and synthesized compounds, along with their percentage yields and physicochemical properties, are shown in Table 1.

Thin-layer chromatography (TLC) and ESI mass spectrometry were used to monitor all the reactions. Purification procedures such as recrystallization and column chromatography with silica gel (100–200 mesh size) and EtOAc in hexane as eluent (10–100%) were used to purify crude products from each step. For structure confirmation, ^1^H-NMR, ^13^C-NMR, and ESI-MS were used. The spectra are given in the Supplementary Materials.

### 2.3. Biological Activity 

#### 2.3.1. HDAC Inhibitor Screening Using HDAC4, HDAC6, and HDAC8 Activity Assay

To assess HDAC6 selectivity, and to better understand the mechanism of action of the newly synthesized compounds, derivatives **5a**–**5s** were tested for their inhibitory activity against HDAC isoenzymes (HDAC4, HDAC6, and HDAC8) with SAHA (vorinostat), Trichostatin A, and Tubastatin as reference drugs using the methods described in the literature and Supplementary Materials [49]. To assess the inhibitory activity of the synthesized compounds against the target enzyme HDAC6, as well as the selectivity against other HDAC isozymes, classic enzyme activity assays were performed using HDAC6 and representatives of class IIa (HDAC4) and class I (HDAC8) HDACs. In the first activity screening, the residual enzyme activity was determined in the presence of 35 µM compound in duplicate to identify the most potent hits (Table 1). All active compounds with a residual activity of less than 50% were retested in a full dose–response series to accurately determine the corresponding IC_50_ value. Most tested compounds showed sub-micromolar activities on HDAC6 with varying selectivity against HDAC4 and HDAC8. Residual enzyme activity is summarized in Figure S1 and Table S1 of the Supplementary Materials.

The most active compound was **5b**, exhibiting an IC_50_ value of 150 nM against HDAC6, while HDAC4 and HDAC8 were inhibited with IC_50_ values of 2300 and 1400 nM, respectively. Activity and selectivity are comparable with the widely used reference compound Tubastatin (Table 2).

The most selective, but still highly active compound was **5o** with an IC_50_ value of 400 nM on HDAC6, and 18-fold and 36-fold selectivity against class IIa HDAC4 and class I HDAC8, respectively. The activity data clearly demonstrate that the scaffold consisting of a quinazoline cap and a benzhydroxamic acid moiety as the linker and zinc-binding group is very useful to design very potent HDAC6 inhibitors. Furthermore, the data show that the selectivity against other HDAC isozymes can be tuned by varying the substitution pattern at R_1_ and R_2_ position, while preserving activity against HDAC6.

#### 2.3.2. Antiproliferative Activity of the Representative Compounds

To access the activity of the synthesized HDAC inhibitors at the cellular level, the antiproliferative impact of the representative compounds was analyzed by the MTT (3-(4,5-dimethylthiazol-2-yl)-2,5-diphenyl tetrazolium bromide) assay. Two human cancer cell lines (HCT116 and MCF7) and the murine B16 melanoma cancer cell line (HEK 293) were treated with the compounds; as shown in Table 3, most of the compounds had an impact on the proliferation rate of all cancer cell lines tested. Notably, compound **5c** exhibited significant cytotoxicity toward all three cancer cell lines. On the basis of these observations, we conclude that compound **5c** exhibited a high antiproliferation effect on multiple human cancer cell lines and was considered for further experiments.

#### 2.3.3. Impact of **5c** on Acetylation of α-Tubulin in MCF7 and HCT-116 Cancer Cell Lines

**Figure 7:**
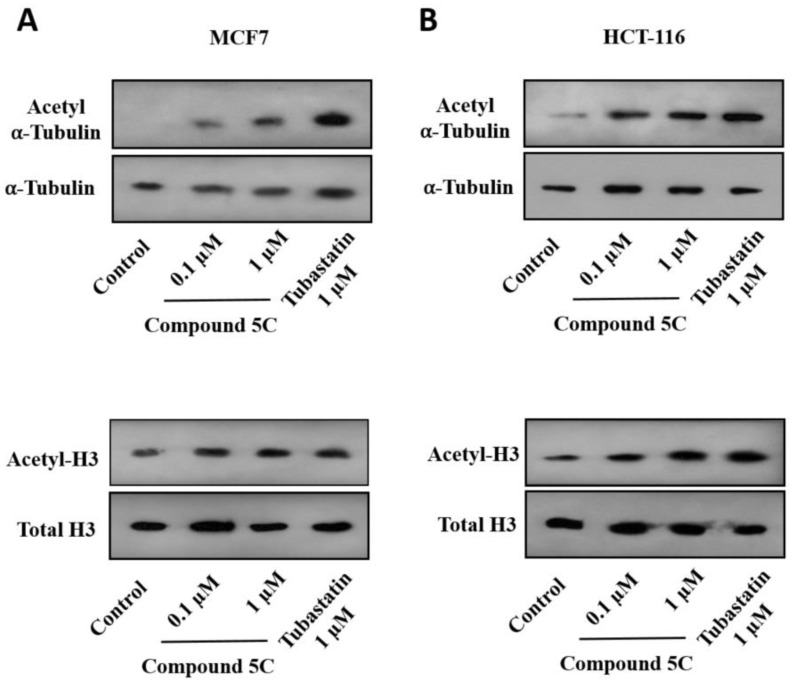
Western blot analysis of acetyl α-tubulin and acetyl-H3 in MCF7 and HCT-116 cells treated with **5c**. Total cell lysate treated with compound **5c** at indicated concentrations and quantified for acetyl α-tubulin (**A**) and acetyl-H3 (**B**) by Western blotting technique.

HDAC6 plays a crucial role in chromatin modulation and deacetylation of α-tubulin, contributing to the expression of oncogenes driving tumorigenesis. Over time, numerous studies have shown HDAC inhibitors (HDACi) as potential anticancer therapeutics [50,51]. The HDAC6-inhibitory effect of compound **5c** was evaluated by analyzing the protein level expression of acetyl α-tubulin in MCF7 and HCT-116 cells treated with **5c** by Western blotting [52]. As shown in Figure 7, there was heightened acetylation of α-tubulin, reflecting the inhibition of HDAC6 activity, starting at 0.1–1 μM of **5c**. Additionally, we noted an increase in acetylation of H3, but only at a higher concentration, to a similar extent to the HDAC6-selective reference compound Tubastatin, as compared to the untreated control, suggesting the potential activity of **5c** at lower concentrations with a limited off-target effect on H3.

#### 2.3.4. Compound **5c** Induces Apoptotic Pathway in MCF7 and HCT-116 Cells

**Figure 8:**
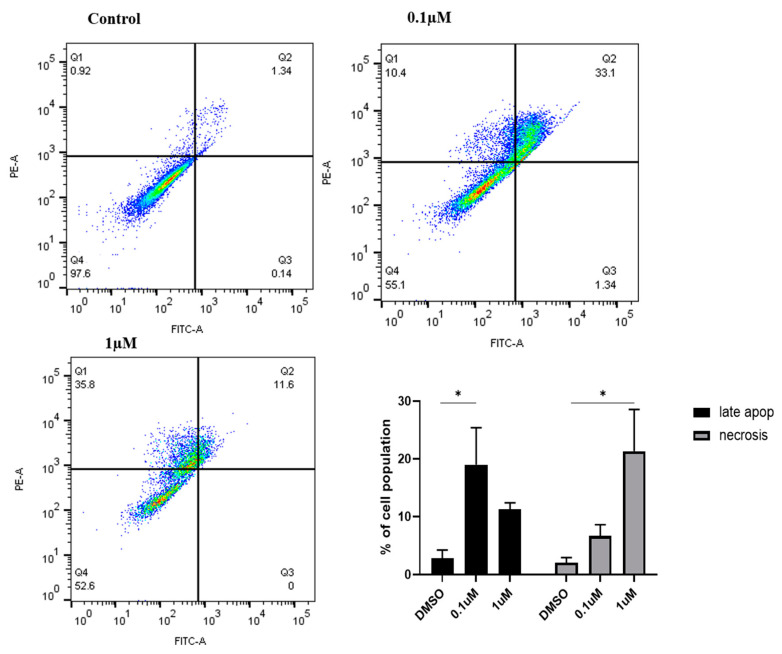
Apoptotic effect of compound **5c** on MCF7 cells. Apoptosis assay using flow cytometry after annexin V–FITC/PI staining of **5c**-treated cells at 0.1 μM and 1 μM concentrations for 24 h. Representative scattered plot of PI vs. annexin V, along with graph depicting the percentage of the population in apoptosis and necrosis state. Data are presented as the mean ± SE of triplicate experiments; * *p* < 0.05.

The efficacy of compound **5c** to induce apoptosis was determined by annexin V–FITC/PI assay using flow cytometry [53]. MCF7 cells were treated with two different concentrations of **5c** as indicated, and the percentages of cells in the apoptotic and necrotic stage were analyzed, as depicted in Figure 8. We noticed that, with the increase in concentration of **5c**, the cells exhibited late apoptosis and necrosis. 

**Figure 9:**
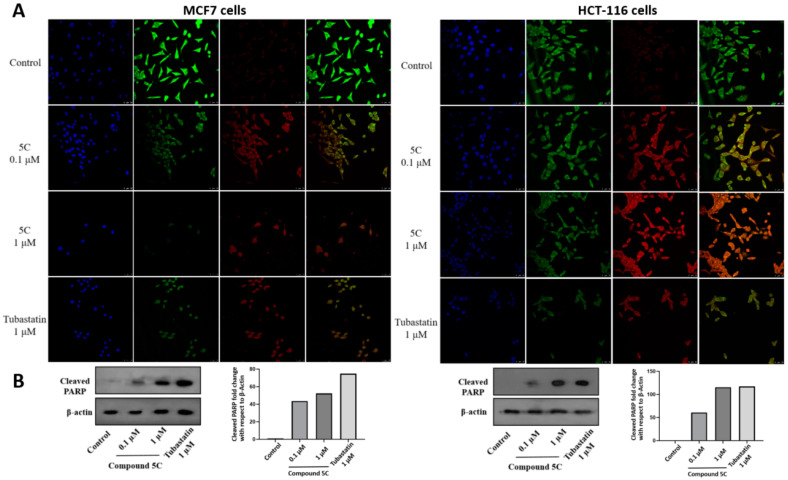
Apoptotic effect of compound **5c** on MCF7 and HCT-116 cells. (**A**) Fluorescence images at 40× magnification of DAPI and AO staining of MCF7 cells (panel on the left) and HCT-116 cells (panels on the right) after treatment with **5c** for 48 h, showing an increase in DNA damage in a dose-dependent manner. The columns show from left to right DAPI staining, AO staining (green channel), AO staining (red channel), and AO staining (merged). (**B**) Western blot analysis of cleaved PARP protein in **5c**-treated MCF7 cells (left panel) and HCT-116 cells (right panel).

Furthermore, acridine orange staining (AO) and fluorescence imaging in MCF7 and HCT-116 cells treated with **5c** indicated evidential apoptosis, as shown in Figure 9A [54]. The control had intact nuclei, as depicted by the green fluorescence due to AO binding to dsDNA, in contrast to the apoptotic nuclei of **5c**-treated cells depicted by orange fluorescence due to AO staining of ssDNA and RNA. This observation was supported by the protein expression of cleaved poly (ADP-ribose) polymerase (PARP), which represents the degree of apoptosis in cells, as shown in Figure 9B. The protein expression of cleaved PARP in **5c** treated MCF7 and HCT-116 cells was significantly higher as compared to untreated cells, supporting the hypothesis that the antiproliferative effect of compound **5c** is majorly contributed by apoptosis. Acridine orange (AO) staining can also be influenced by autophagosomal pH. However, the presence of the DAPI (nuclear localization) signal merged with acridine orange suggests that the conclusions were drawn on the basis of the interaction of AO bound to fragmented or ssDNA in the nucleus.

#### 2.3.5. Impact of Compound **5c** on Cell-Cycle Progression in MCF7 Cells

**Figure 10:**
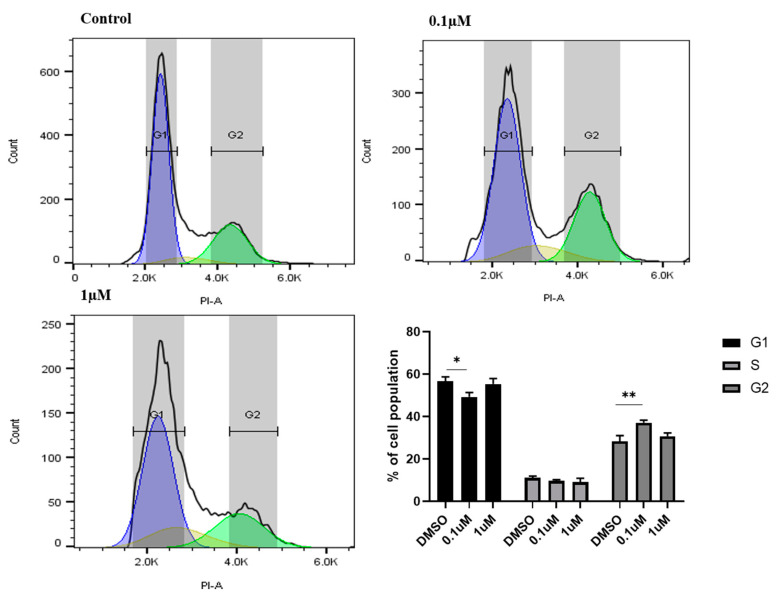
Cell-cycle analysis of MCF7 upon treatment with **5c**. Typical cell-cycle profile and quantified histogram representing the population of cells in each phase upon **5c** treatment for 48 h as compared to the control. Data are presented as the mean ± SE of triplicate experiments; * *p* < 0.05; ** *p* < 0.01.

Cell-cycle distribution analysis was carried out further to evaluate the effect of **5c** on HDAC inhibition, as HDCA inhibitors are mainly known to target genes regulating the cell cycle [55,56]. The **5c**-treated MCF7 cells were subjected to cell-cycle analysis by flow cytometry. A significant increase in the G2/M phase was observed upon treatment with 0.1 μM **5c**, accompanied by a slight reduction in cell population in the G1 and S phases. These observations suggest that treatment with **5c** at 0.1 μM concentration had a better impact on cell proliferation by means of apoptosis and cell-cycle arrest, whereas the 1 μM concentration led to necrosis and cell death (Figure 10). 

#### 2.3.6. Effects of **5c** on Colony-Formation Capability of MCF7 and HCT-116 Cells

**Figure 11:**
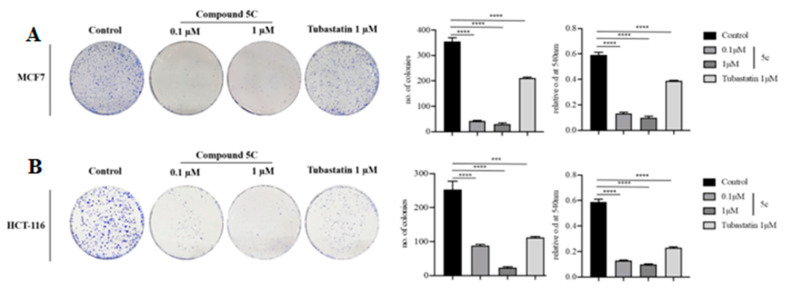
A concentration-dependent impact was observed on the colony formation of MCF7 and HCT-116 cells upon **5c** treatment. (**A**) Images representing the effect of compound **5c** on MCF7 and HCT-116 cells to form colonies. (**B**) Quantified histogram representation of relative colony number and the absorbance of crystal violet stain taken up by the cells, presented as the mean ± SE of triplicate experiments; *** *p* < 0.001, **** *p* < 0.0001.

To evaluate the effect of compound **5c** on the cell survival of MCF7 and HCT-116 cells, a clonogenic assay was performed. Colony formation of MCF7 and HCT-116 cells was inhibited by compound **5c** in a dose-dependent manner, compared to the control cells treated with DMSO (Figure 11) [44]. The number of colonies was significantly reduced by 0.1 μM concentration treatment with **5c**, whereas, at 1 μM concentration, the cell anchorage and growth were almost completely arrested. This further proved that compound **5c** reduced the proliferation and survival rate of cancer cells in vitro.

According to the final results obtained through MTT assay data, we are convinced that compound **5c**, when tested on the representative cancer cell lines and normal cell lines, exhibited a potent IC_50_ compared to known HDAC inhibitors (SAHA, Ricolinostat, Tubastatin, and Trichostatin A). Additionally, compound **5c**, when tested at a 10-fold lower dilution than its IC_50_, still proved effective in reducing the colony-forming ability (Figure 11) of highly aggressive breast (MCF7) and colon cancer (HCT-116) cell lines, inducing cell apoptosis (Figure 8 and Figure 9) and cell-cycle arrest (Figure 10).

### 2.4. In Silico Predicted ADME Studies

Lipinski’s rule of five is a useful guideline in drug discovery that helps assess the likelihood of a compound to have favorable pharmacokinetic properties and oral bioavailability. It helps in the early stages of drug discovery to prioritize compounds with higher chances of success. The rule is not absolute and serves as a general guideline rather than a strict rule. In particular, not all anticancer drugs adhere to Lipinski’s rule of five. Using in silico methods, it is possible to predict the same parameters used to discover new candidate molecules [57,58]. The in silico results for the 19 final analogues were derived as shown in Table S2. The Lipinski rule fitted the predicted parameters of the analogues within the specified range for all parameters including molecular weight, hydrogen bond acceptor, hydrogen bond donor, and rule violation, except the partition coefficient. Regarding the expected results of the final analogues, these compounds would not be expected to have pharmacokinetic issues during drug development. The Swiss ADME tool was used to predict the properties of synthetic derivatives [59]. Judging from the data, the compounds can appear drug-like and exhibit good passive oral absorption (Table S2).

### 2.5. Molecular Docking

At first, the docking procedure was validated by redocking the respective ligand into the binding pocket of the corresponding crystal structure of the ligand–HDAC complex. In this study, crystal structures 4CBY (HDAC4), 5EDU (HDAC6), and 1T69 (HDAC8) were obtained from the Protein Data Bank, the single global archive for 3D macromolecular structure data [60]. Redocking of the ligand into the crystal structure of the binding pocket of HDAC4 (PDB ID: 4CBY) showed perfect overlap between the docked ligand and the X-ray binding pose (Figure S2A). Redocking into HDAC6 (PDB ID: 5EDU) showed excellent agreement between the crystallized and docked ligand in the lower part of the binding pocket, but considerable deviation in the head group, which can be explained by the fact that this moiety protrudes freely into solution (Figure S2B). Redocking of the ligand into the crystal structure of the binding pocket of HDAC8 (PDB ID: 1T69) showed very good overlap between the docked ligand and the X-ray binding pose in the lower part of the binding pocket (Figure S2C). The phenyl head group, which protrudes into free solution, showed more deviation due to a higher degree of freedom. In summary, the applied procedures were suitable for meaningful docking of ligands into the binding pockets of all three HDAC isozymes. 

**Figure 12:**
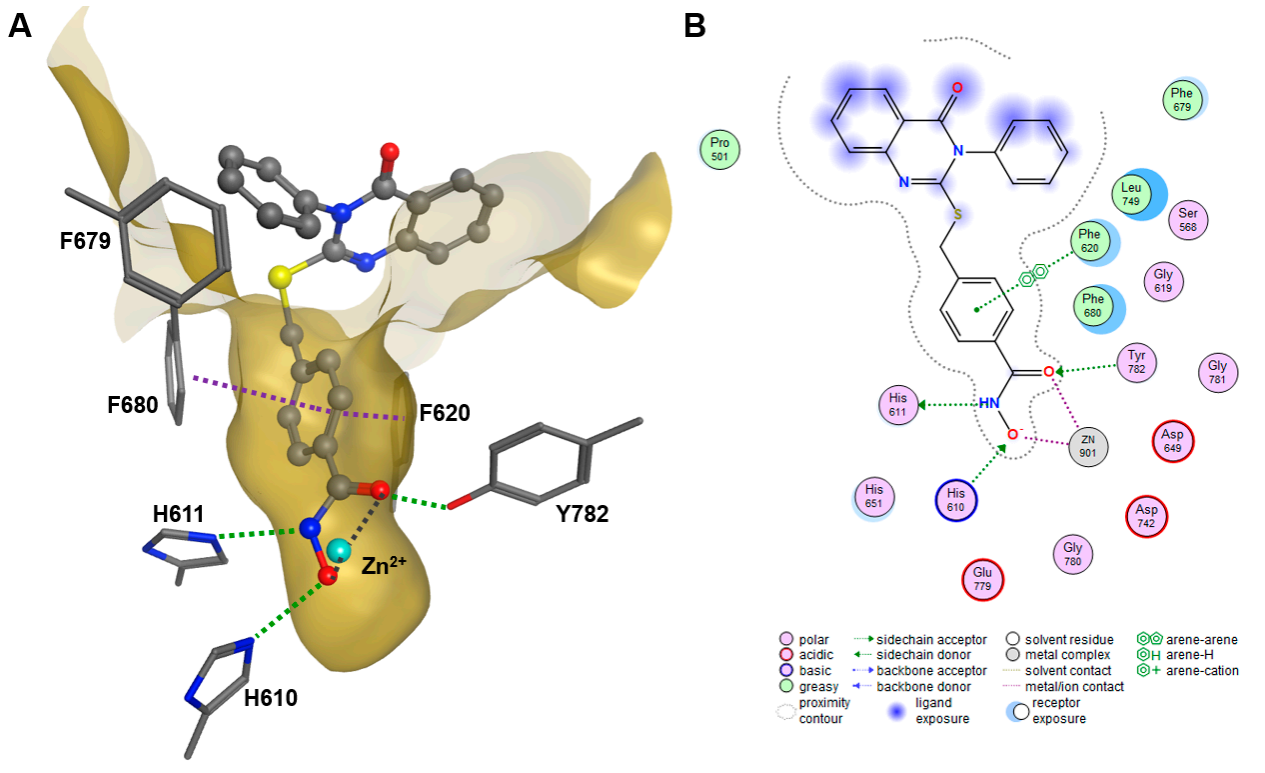
Binding pose of **5b** docked into the crystal structure of HDAC6 (PDB ID: 5EDU). (**A**) The 3D structure showing metal binding (dark-gray dotted line), hydrogen bonds (green dotted lines), and pi–pi stacking (magenta dotted lines). The surface of the binding pocket is illustrated by a transparent ochre plane. (**B**) The 2D interactions between **5b** and HDAC6 binding pocket.

The final compounds, **5a**–**s**, showed clear preference for HDAC6 with IC_50_ values ranging from 150 nM to 5.6 µM. The most potent compound (**5b**), the compound with the lowest HDAC6 activity (**5a**), and the compound with the highest cytotoxicity (**5c**) were chosen for docking against HDAC6 (PDB ID: 5EDU), as well as HDAC4 (PDB ID: 4CBY) and HDAC8 (PDB ID: 1T69). Superpositions of the binding poses of compounds **5a**–**c** in the active site pocket of these HDAC isozymes demonstrated highly overlapping and defined positions of the benzhydroxamate moiety inside the narrow binding channels of the crystal structures of HDAC6 and HDAC8, while there was much larger variation in the binding poses of **5a**–**c** in the widened binding pocket of HDAC4 (Figure S3). Interestingly, the quinazoline head groups of **5a**–**c** also showed excellent overlap, but only when docked into HDAC6 (Figure S3B). Thus, the binding pose of the final compounds in the active site pocket of HDAC6 appeared to be well defined, in agreement with the high, mostly sub-micromolar, activities against this isozyme. More negative docking scores correlate with high activity, but, as usual, cannot predict smaller variations within an order of magnitude. However, docking results confirmed the general selectivity of final compounds against HDAC4 and HDAC8, with generally better GBVI/WSA dG scores for HDAC6 (Table S3). A closer look at the energy-minimized docking pose of **5b** in complex with HDAC6 revealed several strong directed interactions including bidentate chelation of the catalytic zinc ion by the hydroxamate warhead at the bottom of the active site, and three classic hydrogen bonds with catalytically acting amino acids His610, His611, and Tyr782 (Figure 12). The binding was further strengthened by pi-stacking of the benzhydroxamic acid with Phe620 and possibly Phe680, which line the active site binding tunnel. The capping group seemed to form weaker interactions with the surface of HDAC6, while R^1^ substituents were in proximity to Phe679, allowing for hydrophobic interactions, which might explain the observed relatively small differences in activity among **5**-series compounds.

## 3. Materials & Methods

### 3.1. Chemistry

All chemical reagents and solvents were purchased from Aldrich, Alfa Aesar, Finar, India. The solvents and reagents were of LR grade. All the solvents were dried and distilled before use. Thin-layer chromatography (TLC) was carried out on aluminum-supported silica gel plates (Merck 60 F254, Merck KGaA, Darmstadt, Germany) with visualization of components by UV light (254 nm). Column chromatography was carried out on silica gel (Merck 100–200 mesh). ^1^H-NMR and ^13^C-NMR spectra were recorded at 400 MHz and 101 MHz, respectively, using a Bruker AV 400 spectrometer (Bruker Co., Zurich, Switzerland) in CDCl_3_ and DMSO-*d_6_* solution with tetramethylsilane as the internal standard; chemical shift values (δ) were given in ppm. ^1^H NMR spectra were recorded in CDCl_3_ or DMSO-*d_6_*. The following abbreviations are used to designate multiplicities: s = singlet, d = doublet, t = triplet, m = multiplet, and br = broad. Melting points were determined on an electrothermal melting point apparatus (Stuart-SMP30) in open capillary tubes and were uncorrected. Elemental analyses were performed by Elementar Analysensysteme GmbH vario MICRO cube CHN Analyzer. Mass spectra (ESI-MS) were recorded on a Schimadzu LC–MS 8040 MS/ESI mass spectrometer.

### 3.2. General Synthesis Procedure for the Synthesis of ***5a**–**5s***

**Step 1.** Synthesis of intermediates **2a**–**2s**: An oven-dried, 50 mL, round-bottom flask was consecutively charged with a mixture of substituted anthranilic acid (**1a**–**c**) (1 equiv.), different isothiocyanates (**R^2^ a**–**s**) (1.1 equiv.), and triethylamine (2 equiv.) as the base in 35 mL of absolute ethanol. The mixture was heated at reflux for (12 h) in an oil bath. TLC (TLC Silica gel 60 F*_254_*) analysis with petroleum ether/ethyl acetate (7:3) revealed that the reaction was complete. The reaction mixture was cooled to room temperature; the obtained precipitate was separated by filtration. Then, the filtrate was concentrated in vacuo to obtain the intermediates **2a**–**2s**. In Step 1, the total percentage yield was revealed to be in the average range of 85–93% for intermediates (**2a**–**2s**). **Step 2.** Synthesis of intermediates **3a**–**3s**: To a mixture of intermediates **2a**–**2s** (1 equiv.) and methyl 4-(bromomethyl) benzoate (1.05 equiv.) in butanone (25 mL), KI (1 equiv.) and K_2_CO_3_ (3 equiv.) were added; then, the reaction was heated at 75 °C for 4 h. The reaction was monitored for completion by TLC (TLC Silica gel 60 F*_254_*) with petroleum ether/ethyl acetate (8:2). After completion, the solvent was evaporated under vacuo, and the residue was dissolved in water and ethyl acetate. The organic layers were collected, dried over anhydrous Na_2_SO_4_, and concentrated in vacuum. The resultant crude product was purified by crystallization to get the intermediates **3a**–**3s**. Step 2 found that the total percentage yield for intermediates **3a**–**3s** was in the average range of 74–80%. **Step 3.** Synthesis of intermediates **4a**–**4s**: The intermediates **3a**–**3s** containing ester groups were converted to acid intermediates **4a**–**s**. Compounds **3a**–**3s** (1 equiv.) were dissolved in 30 mL of 1,4-dioxane; then, sodium hydroxide NaOH (5 equiv.) was added to this mixture. The reaction was heated at 80 °C for 4 h, in an oil bath. Upon cooling to ambient temperature, the reaction mixture was concentrated in vacuo, and was acidified with 2 N HCl (pH at 3–4) to form precipitation. The precipitate was isolated by filtration and dried to afford intermediates **4a**–**4s**. Step 3 found that the total percentage yield for intermediates **4a**–**4s** was in the average range of 70–80%. **Step 4.** Synthesis of title compounds **5a**–**5s**: The crude compounds **4a**–**4s**)(1 equiv.) were dissolved in DMF (10 mL); then, N,N-diisopropylethylamine (DIPEA) (5 equiv.) as a base and O-benzotriazole-1-yl-N,N,N′,N′-tetramethyl-uroniumhexafluorophosphate (HBTU) (2 equiv.) as the coupling agent were added. The mixture was stirred at 25 °C for 30 min. Afterward, hydroxylamine hydrochloride (NH_2_OH·HCl) (15 equiv.) was added to the solution, before stirring for another 4 h. After completion of the reaction, the mixture was poured into ice water. The system was washed with saturated NaHCO_3_ and brine, and then extracted with ethyl acetate. The organic layers were dried over anhydrous Na_2_SO_4_ and concentrated in vacuo. The product was further purified by column chromatography with an appropriate ethyl acetate/petroleum ether mixture to provide the title compounds **5a**–**5s**.

### 3.3. Enzyme Activity Assay

Full-length recombinant HDAC6 was purchased from BPS Bioscience. HDAC4 and HDAC8 were recombinantly produced in *E. coli* BL21(DE3) using a pET14b vector containing either the codon-optimized catalytic domain of HDAC4 (T648-T1057, full length: UniProt P56524) or codon-optimized full-length HDAC8. To determine IC_50_ values, a serial inhibitor dilution in assay buffer (25 mM Tris-HCl, 75 mM KCl, 0.00001% Pluronic F-127, pH 8.0) was incubated with HDAC in a black 96-well microtiter half-area plate (Greiner, Kremsmünster, Austria) for 1 h at 30 °C. After incubation, the enzymatic HDAC reaction was initiated by adding 20 µM Boc-Lys(trifluoroacetyl)-AMC (Bachem, Bubendorf, Switzerland) as the substrate for HDAC4 and HDAC8, and 50 µM Boc-Lys(acetyl)-AMC (Bachem) as the substrate for HDAC6. After incubation for 1 h at 30 °C, the reaction of HDAC4 and HDAC8 was stopped by adding 1.7 µM of suberoylanilinetrifluoromethylketone (SATFMK), while the reaction of HDAC6 was stopped by adding 4.2 µM of suberoylanilidehydroxamic acid (SAHA). By addition of 0.4 mg/mL trypsin (AppliChem, Darmstadt, Germany), the deacetylated substrates were converted into a fluorescent product. Respective fluorescence intensities were measured in a microplate reader (PheraStar Plus, BMG Labtech, Ortenberg, Germany) at 450 nm (Ex: 350 nm) and correlated to enzyme activity. Dose–response data were plotted and analyzed using GraphPad Prism 6 software and fitted to a four-parameter logistic fit [49].

(1) $$EA=E_{0}+\frac{\left(E_{max}-E_{0}\right)}{1+10^{\left(\log\left(IC_{50}\right)-x\right)\times h}},$$

where *EA* is the enzyme activity at a given inhibitor concentration *x*, *E_max_* and *E*_0_ are the enzyme activities determined at zero and complete inhibition, respectively, *h* is the slope of the curve, and *IC*_50_ indicates the inhibitor concentration at which half of the enzyme is inhibited.

### 3.4. Cell Culture

The human cancer cell lines HCT-116, MCF7, and HEK293, as well as murine B16 melanoma cancer cells, were grown in Dulbecco modified Eagle’s medium (DMEM) with 10% fetal bovine serum (FBS) and 1% penicillin/streptomycin antibiotics in a humidified incubator with 5% CO_2_ at 37 °C.

### 3.5. Cell Proliferation Assay

For cell viability and proliferation, the MTT assay was performed. Cells were seeded at a concentration of 2000 cells/100 µL medium in a 96-well plate; once adhered, they were treated with various concentrations of representative compounds, along with Erlotinib, which is a commercially used cancer therapeutic, HDAC inhibitors like SAHA, Ricolinostat, Tubastatin, and Trichostatin A, and then incubated for 72 h. This was followed by cell viability analysis using MTT according to the manufacturer’s instructions. Briefly, 10 μL of MTT solution was added to each well and incubated for 1–4 h at 37 °C. The formazan crystals formed were dissolved in 200 μL of solubilization solution (DMSO), and absorbance was recorded at 570 nm. Graphs were plotted according to the absorbance value, and the half-maximal inhibitory concentration (IC_50_) value was determined with the help of GraphPad Prism 8.0.2.

### 3.6. Western Blotting

On the basis of the results obtained from the cell proliferation assay, further experiments were conducted on MCF7 and HCT-116 cells treated with compound **5c**. The cells were harvested, and 0.1 × 10^6^ cells were seeded in a 12-well cell culture plate and allowed to adhere properly. The cells were then incubated with 0.1 μM and 1 μM concentrations of compound **5c** for 24 h. Cells were pelleted in 1× PBS and lysed using ice-cold RIPA buffer. The protein concentration of the lysate was quantified using the Bradford assay and an equal concentration of sample proteins was run on 12% SDS-PAGE and transferred onto 0.45 μm PVDF membrane (Millipore (St. Louis, MO, USA), Immobilon IPVH00010). The membrane was blocked with 5% skimmed milk in 1× TBST and probed with primary antibodies of acetyl-H3 (CST (Danvers, MA, USA), 9677S), total H3 (CST, 9715S), acetyl-α tubulin (CST, 5335T), total α-tubulin (CST, 2125S), cleaved PARP (CST, 5625T), and β-actin (Sigma Aldrich (Burlington, MA, USA), A3854) overnight at 4 °C and HRP-conjugated secondary antibody (Jackson Immuno research Laboratories (West Grove, PA, USA), 111-035-003) for 4–5 h in 4 °C. After thorough washing, the protein signals were visualized using enhanced chemiluminescence (ECL) kit (Bio-Rad (Hercules, CA, USA), 1705061) and captured onto an X-ray sheet in a dark room [52]. 

### 3.7. Cell Apoptosis Analysis

The apoptosis assay was performed using an annexin V–FITC Apoptosis detection kit (Sigma-Aldrich, APOAF) according to the manufacturer’s instructions. Cells were harvested, and 0.1 × 10^6^ cells were seeded in a six-well cell culture plate and allowed to adhere followed by treatment with DMSO as the control and compound **5c** at 0.1 μM and 1 μM concentrations for 24 h. The cells were trypsinized, pelleted down, and resuspended in the 1× buffer provided. The cells were stained using annexin V–FITC/PI and analyzed immediately using flow cytometry. Further analysis and plotting of graphs were conducted using Flowjo 7.6 [53]. 

### 3.8. Cell-Cycle Analysis

Cell-cycle analysis was carried out using a cell-cycle analysis kit (Sigma-Aldrich, MAK344) according to the protocol given by the manufacturer. MCF7 cells were treated with DMSO or 0.1 μM and 1 μM concentrations of compound **5c** and incubated for 48 h. Cells were then trypsinized and pelleted down. The pellets were washed with 1× cell-cycle buffer and fixed in ice-cold 70% ethanol for 1 h at 4 °C. The fixated cells were treated with ribonuclease A (RNase A) and stained with PI at room temperature for 30 min. Analysis was carried out using FlowJo 10.7.2. [55]. 

### 3.9. Clonogenic Assay

MCF7 and HCT-116 cells were seeded in a six-well culture plate at a density of 500 cells/well. After cells adhered completely, they were incubated in the culture medium with DMSO, 0.1 μM **5c**, and 1 μM **5c**. The medium, along with the treatment, was replenished every 3 days for 12–14 days. The colonies formed were fixed with a 3:1 ratio of methanol and glacial acetic acid for 10 min. The wells were then washed with 1× PBS, and cells were stained with 0.5% *v*/*v* crystal violet solution for 10 min [55]. Excess stain was washed off in running water, and images were taken after proper drying. Colonies were counted visually or using ImageJ software, NIH (Version 1.53K). The stain taken up by the colonies was dissolved in 0.1% SDS, and absorbance was read at 540 nm. Graphs were plotted using GraphPad Prism 8.0.2. 

### 3.10. Statistical Analysis

The data were expressed as the mean ± SE. Statistical tests were performed using GraphPad Prism Software version 8.0.2. Comparisons of the observed data were analyzed by one-way ANOVA. The statistical significance was set at * *p* < 0.05, ** *p* < 0.01, *** *p* < 0.001, and **** *p* < 0.0001.

### 3.11. Molecular Docking

Modeling, preparation, and visualization of structural data, as well as molecular docking, were performed using MOE 2020.0901 software (Chemical Computing Group ULC, Montreal, QC, Canada). Three crystal structures, PDB IDs 4CBY (HDAC4), 5EDU (HDAC6), and 1T69 (HDAC8), were obtained from the RCSB Protein Data Bank and subjected to the Quickprep procedure of MOE 2019, including 3D protonation for subsequent docking. The partial charges of all protein and ligand atoms were calculated using the implemented Amber14 force field. The docking site was defined by the ligand within the binding pocket of the respective crystal structure. Molecular docking was performed choosing the triangle matcher for placement of the ligand in the binding site and ranked with the London dG scoring function. The best 50 poses were passed onto refinement and energy minimization in the pocket using the induced fit method, and the 10 best poses were rescored using the GBVI/WSA dG scoring function.

### 3.12. Analytical Data for the Final Compounds (***5a**–**5s***)

***4-(((3-(4-chlorophenyl)-4-oxo-3,4-dihydroquinazolin-2-yl)thio)methyl)-N-hydroxybenzamide (5a):***

Off-white solid; yield 66%, m.p. 155–160 °C. ^1^H-NMR (400 MHz, DMSO-*d_6_*) δ 11–8.50 (bs, 1H), δ 8.08 (d, *J* = 7.7 Hz, 1H), 7.85 (dd, *J* = 8.9, Hz, 1H), 7.75–7.68 (s, 1H), 7.66–7.59 (m, 4H), 7.56–7.45 (m, 4H), 7.40 (dd, *J* = 9.5, 7.7 Hz, 1H), 4.41 (s, 2H). ^13^C-NMR (101 MHz, DMSO-*d_6_*) δ 167.75, 164.79, 161.19, 156.77, 147.83, 139.94, 135.88, 134.64, 131.90, 129.95, 128.96, 127.39, 126.37, 120.01, 35.85. ESI-MS: (*m*/*z*) calculated for C_22_H_16_ClN_3_O_3_S: 437.06; found: 438 (M + H)^+^; analysis calculated (%): C, 60.34; H, 3.68; Cl, 8.10; N, 9.60; O, 10.96; S, 7.32; found: C, 60.30; H, 3.72; Cl, 8.20; N, 9.50; O, 10.95; S, 7.33.

***N-hydroxy-4-(((4-oxo-3-phenyl-3,4-dihydroquinazolin-2-yl)thio)methyl)benzamide (5b):***

Off-white solid; yield 52%, m.p. 160–166 °C. ^1^H-NMR (400 MHz, DMSO-*d_6_*) δ 8.08 (dd, *J* = 7.9, 1.2 Hz, 1H), 7.90–7.86 (m, 3H), 7.84 (d, *J* = 1.5 Hz, 1H), 7.70 (d, *J* = 7.8 Hz, 1H), 7.63–7.57 (m, 1H), 7.57–7.52 (m, 3H), 7.52–7.41 (m, 3H), 4.47 (s, 2H). ^13^C-NMR (101 MHz, DMSO-*d_6_*) δ 166.41, 159.11, 156.80, 147.83, 145.81, 136.30, 135.50, 130.41, 130.17, 130.01, 129.85, 129.56, 128.89, 126.96, 126.59, 120.37, 119.04, 35.94. ESI-MS: (*m*/*z*) calculated for C_22_H_17_N_3_O_3_S: 403.10; found: 404 (M + H)^+^; analysis calculated (%): C, 65.49; H, 4.25; N, 10.42; O, 11.90; S, 7.95; found: C, 65.45; H, 4.29; N, 9.42; O, 12.90; S, 7.95.

***N-hydroxy-4-(((4-oxo-3-(p-tolyl)-3,4-dihydroquinazolin-2-yl)thio)methyl)benzamide (5c):***

White solid; yield 65%, m.p. 138–142 °C. ^1^H-NMR (400 MHz, DMSO-*d_6_*) δ 11.81–9.11 (bs, 1H). δ 8.08 (dd, *J* = 7.9, 1.1 Hz, 1H), 7.89–7.80 (m, 2H), 7.66 (dt, *J* = 7.4, 11.8 Hz, 2H), 7.54–7.42 (m, 3H), 7.39–7.21 (m, 4H), 4.61 (s, 2H) 2.38 (s, 3H). ^13^C-NMR (101 MHz, DMSO-*d_6_*) δ 164.46, 162.82, 161.24, 157.60, 147.65, 140.86, 140.08, 135.41, 133.60, 132.22, 130.46, 129.96, 129.56, 127.38, 127.06, 126.51, 120.05, 35.57, 21.46. ESI-MS: (*m*/*z*) calculated for C_23_H_19_N_3_O_3_S: 417.11; found: 418 (M + H)^+^; analysis calculated (%): C, 66.17; H, 4.59; N, 10.07; O, 11.50; S, 7.68; found: C, 65.10; H, 5.54; N, 10.15; O, 11.41; S, 6.70.

***4-(((3-(4-ethylphenyl)-4-oxo-3,4-dihydroquinazolin-2-yl)thio)methyl)-N-hydroxybenzamide (5d):***

Off-white solid; yield 64%, m.p. 150–157 °C. ^1^—NMR (400 MHz, DMSO-*d_6_*) δ 11.35 (bs, 1H), δ 8.07 (d, *J* = 7.9 Hz, 1H), 7.93 (dd, *J* = 8.1, 1.3 Hz, 1H), 7.86 (t, *J* = 7.7 Hz, 1H), 7.69 (ddd, *J* = 10, 10.3, 6.3 Hz, 1H), 7.49 (m, 2H), 7.37 (m, 3H), 7.34–7.29 (m, 3H), 3.35 (s, 2H), 2.75 (q, 2H), 1.32 (t, 3H). ^13^C-NMR (101 MHz, DMSO-*d_6_*) δ 170.83, 150.20, 150.06, 147.04, 144.28, 135.36, 132.91, 129.97, 129.28, 128.29, 127.75, 115.66, 114.57, 31.28, 28.32, 13.48. ESI-MS: (*m*/*z*) calculated for C_24_H_21_N_3_O_3_S: 431.13; found: 432 (M + H)^+^; analysis calculated (%): C, 66.80; H, 4.91; N, 9.74; O, 11.12; S, 7.43; found: C, 66.85; H, 4.86; N, 9.72; O, 11.10; S, 7.47.

***4-(((3-(4-bromophenyl)-4-oxo-3,4-dihydroquinazolin-2-yl)thio)methyl)-N-hydroxybenzamide (5e):***

Pale-yellow solid; yield 58%, m.p. 170–175 °C. ^1^H-NMR (400 MHz, DMSO-*d_6_*) δ 11.69–10.15 (bs, 1H), 8.95 (s, 1H).8.95 (d, *J* = 8.5 Hz, 1H), 8.08 (d, *J* = 7.7 Hz, 1H), 7.87 (t, *J* = 7.4 Hz, 1H), 7.75 (t, *J* = 9.2 Hz, 2H), 7.69 (dd, *J* = 9.51, 8.1 Hz, 2H), 7.59–7.35 (m, 4H), 4.45 (s, 2H), ^13^C-NMR (101 MHz, DMSO-*d_6_*) δ 164.78, 161.04, 156.58, 147.52, 140.67, 135.55, 133.05, 132.17, 131.88, 129.82, 127.40, 127.06, 126.58, 123.80, 119.75, 35.85. ESI-MS: (*m*/*z*) calculated for C_22_H_16_BrN_3_O_3_S: 481.01; found: 484 (M + H)^+^; analysis calculated (%): C, 54.78; H, 3.34; Br, 16.57; N, 8.71; O, 9.95; S, 6.65; found: C, 54.77; H, 3.35; Br, 16.55; N, 8.72; O, 9.93; S, 6.67.

***4-(((3-(3,4-dimethylphenyl)-4-oxo-3,4-dihydroquinazolin-2-yl)thio)methyl)-N-hydroxybenzamide (5f):***

White solid; yield 63%, m.p. 138–144 °C. ^1^H-NMR (400 MHz, DMSO-*d_6_*) δ 8.80 (bs, 1H), 8.12–8.02 (m, 1H), 7.90–7.80 (m, 1H), 7.76–7.55 (m, 1H), 7.55–7.40 (m, 2H), 7.37–7.21 (m, 3H), 7.14 (dd, *J* = 9.5, 11.3 Hz, 2H), 4.56 (s, 2H), 2.27 (s, 3H) 2.29 (s, 3H). ^13^C-NMR (101 MHz, DMSO-*d_6_***)** δ 164.46, 161.47, 157.41, 147.61, 143.03, 139.13, 138.24, 135.47, 133.74, 130.80, 130.22, 129.65, 127.13, 127.04, 126.94, 126.54, 126.47, 120.02, 36.04, 19.74. ESI-MS: (*m*/*z*) calculated for C_24_H_21_N_3_O_3_S: 431.13; found: 432 (M + H)^+^; analysis calculated (%): C, 66.80; H, 4.91; N, 9.74; O, 11.12; S, 7.43; found: C, 66.80; H, 4.97; N, 9.70; O, 11.16; S, 6.40.

***N-hydroxy-4-(((4-oxo-3-(m-tolyl)-3,4-dihydroquinazolin-2-yl)thio)methyl)benzamide (5g):***

Off-white solid; yield 65%, m.p. 137–144 °C. ^1^H-NMR (400 MHz, DMSO-*d_6_*) δ 11.31–8.46 (bs, 1H), 8.08 (dd, *J* = 7.9, 1.1 Hz, 1H), 7.94 (d, *J* = 12.2 Hz, 1H), 7.90–7.82 (m, 1H), 7.71 (d, *J* = 8.0 Hz, 1H), 7.66 (d, *J* = 8.2 Hz, 1H), 7.56–7.46 (m, 3H), 7.43 (t, *J* = 7.7 Hz, 2H), 7.38–7.30 (m, 1H), 7.28–7.16 (m, 2H), 4.55 (s, 2H), 2.36 (s, 3H). ^13^C-NMR (101 MHz, DMSO-*d_6_*) δ 164.70, 160.83, 157.49, 147.62, 141.01, 140.79, 139.58, 139.49, 136.13, 135.44, 132.51, 132.17, 131.02, 130.05, 129.81, 127.40, 127.04, 126.82, 126.19, 119.92, 36.01, 20.38. ESI-MS: (*m*/*z*) calculated for C_23_H_19_N_3_O_3_S: 417.11; found: 418 (M + H)^+^; analysis calculated (%): C, 66.17; H, 4.59; N, 10.07; O, 11.50; S, 7.68; found: C, 66.10; H, 4.66; N, 10.02; O, 11.55; S, 7.62.

***4-(((3-(3-chlorophenyl)-4-oxo-3,4-dihydroquinazolin-2-yl)thio)methyl)-N-hydroxybenzamide (5h):***

Off-white solid; yield 70%, m.p. 136–140 °C. ^1^H-NMR (400 MHz, DMSO-d_6_) δ 9.94 (bs, 1H), 8.08 (d, *J* = 6.7 Hz, 1H), 7.88 (dd, *J* = 9.6, 18.9 Hz, 1H), 7.64 (m, 4H), 7.50 (m, 4H), 7.28 (d, *J* = 10.5 Hz, 2H), 4.46 (s, 2H). ^13^C-NMR (100 MHz, DMSO-*d_6_*) δ 169.19, 162.21, 159.68, 146.65, 140.53, 140.49, 134.45, 133.86, 133.38, 129.85, 129.56, 129.56, 128.56, 128.05, 127.53, 127.53, 126.74, 126.63, 125.97, 124.61, 117.74, 37.07. ESI-MS: (*m*/*z*) calculated for C_22_H_16_ClN_3_O_3_S: 437.06; found: 438 (M + H)^+^; analysis calculated (%): C, 60.34; H, 3.68; Cl, 8.10; N, 9.60; O, 10.96; S, 7.32; found: C, 60.32; H, 3.72; Cl, 8.15; N, 9.55; O, 10.90; S, 7.34.

***4-(((3-(2-fluorophenyl)-4-oxo-3,4-dihydroquinazolin-2-yl)thio)methyl)-N-hydroxybenzamide (5i):***

White solid; yield 54%, m.p. 130–136 °C. ^1^H-NMR (400 MHz, DMSO-*d_6_*) δ 8.90–8.70 (m, 1H), 8.10 (d, *J* = 7.6 Hz, 1H), 7.98–7.83 (m, 1H), 7.79–7.69 (m, 1H), 7.69–7.57 (m, 2H), 7.45 (m, 3H), 7.34–7.23 (m, 4H), 7.18–6.99 (m, 1H), 6.46 (d, *J* = 8.3 Hz, 1H), 4.59 (s, 2H). ^13^C-NMR (101 MHz, DMSO-*d_6_*) δ 163.79, 162.84, 160.49, 156.74, 147.23, 135.94, 135.81, 133.02, 132.08, 131.86, 130.58, 129.53, 127.11, 126.95, 126.71, 125.97, 119.47, 117.18, 116.99, 114.24, 35.84. ESI-MS: (*m*/*z*) calculated for C_22_H_16_FN_3_O_3_S: 421.09; found: 422 (M + H)^+^; analysis calculated (%): C, 62.70; H, 3.83; F, 4.51; N, 9.97; O, 11.39; S, 7.61; found: C, 62.75; H, 3.85; F, 4.50; N, 9.98; O, 11.35; S, 7.66.

***4-(((7-chloro-4-oxo-3-phenyl-3,4-dihydroquinazolin-2-yl)thio)methyl)-N-hydroxybenzamide (5j):***

Pale-yellow solid; yield 62%, m.p. 229–236 °C. ^1^H-NMR (400 MHz, DMSO-*d_6_*) δ 9.44 (bs, 1H), 8.07 (d, *J* = 8.5 Hz, 1H), 7.77 (dd, *J* = 9.6, 5.5 Hz, 1H), 7.71–7.59 (m, 2H), 7.52 (dd, *J* = 10.4, 3.4 Hz, 4H), 7.40 (m, 4H), 4.60 (s, 2H). ^13^C-NMR (100 MHz, DMSO-*d_6_*) δ 169.10, 162.25, 159.25, 145.62, 140.49, 140.20, 137.21, 133.86, 133.34, 132.34, 129.89, 128.89, 125.56, 129.56, 127.37, 128.31, 127.53, 127.53, 123.82, 121.14, 118.10, 36.08. ESI-MS: (*m*/*z*) calculated for C_22_H_16_ClN_3_O_3_S: 437.06; found: 438 (M + H)^+^; analysis calculated (%): C, 60.34; H, 3.68; Cl, 8.10; N, 9.60; O, 10.96; S, 7.32; found: C, 60.32; H, 3.72; Cl, 8.11; N, 9.60; O, 10.90; S, 7.38.

***4-(((7-chloro-4-oxo-3-(o-tolyl)-3,4-dihydroquinazolin-2-yl)thio)methyl)-N-hydroxybenzamide (5k):***

White solid; yield 63%, m.p. 180–180 °C. ^1^H-NMR (400 MHz, DMSO-*d_6_*) δ 7.84–7.70 (m, 2H), 7.48 (d, *J* = 6.2 Hz, 2H), 7.38–7.29 (m, 2H), 7.25 (d, *J* = 10.4 Hz, 2H), 7.09 (s, 1H), 6.96 (d, *J* = 9.1 Hz, 2H), 6.88 (s, 1H), 4.29–4.25 (m, 2H), 2.33–2.29 (s, 3H). ^13^C-NMR (100 MHz, DMSO-*d_6_*) δ 169.19, 162.10, 161.12, 145.62, 140.49, 140.20, 136.73, 135.84, 133.86, 131.15, 130.44, 129.90, 129.56, 129.56, 128.31, 127.67, 127.53, 123.82, 119.10, 37.07, 17.23. ESI-MS: (*m*/*z*) calculated for C_23_H_18_ClN_3_O_3_S: 451.08; found: 452 (M + H)^+^; analysis calculated (%): C, 61.13; H, 4.01; Cl, 7.84; N, 9.30; O, 10.62; S, 7.09; found: C, 61.15; H, 4.02; Cl, 7.80; N, 9.10; O, 9.62; S, 8.09.

***4-(((7-chloro-4-oxo-3-(p-tolyl)-3,4-dihydroquinazolin-2-yl)thio)methyl)-N-hydroxybenzamide (5l):***

White solid; yield 59%, m.p. 170–175 °C. ^1^H-NMR (400 MHz, DMSO-*d_6_*) δ 8.07 (d, *J* = 9.2 Hz, 1H), 7.88 (d, *J* = 8.2 Hz, 2H), 7.78 (d, *J* = 1.8 Hz, 1H), 7.60 (d, *J* = 8.2 Hz, 2H), 7.50 (dd, *J* = 8.5, 1.9 Hz, 1H), 7.41–7.27 (m, 4H), 4.46 (s, 2H), 2.39 (s, 3H). ^13^C-NMR (101 MHz, DMSO-*d_6_*) δ 162.79, 152.28, 145.20, 139.36, 137.50, 134.28, 129.79, 129.65, 129.15, 121.50, 116.88, 114.01, 36.55, 20.71. ESI-MS: (*m*/*z*) calculated for C_23_H_18_ClN_3_O_3_S: 451.08; found: 452 (M + H)^+^; analysis calculated (%): C, 61.13; H, 4.01; Cl, 7.84; N, 9.30; O, 10.62; S, 7.09; found: C, 61.15; H, 4.02; Cl, 7.82; N, 9.35; O, 10.60; S, 8.05.

***4-(((7-chloro-3-(2-fluorophenyl)-4-oxo-3,4-dihydroquinazolin-2-yl)thio)methyl)-N-hydroxybenzamide (5m):***

White solid; yield 60%, m.p. 151–156 °C. ^1^H-NMR (400 MHz, DMSO-*d_6_*) δ 7.96 (s, 1H), 7.86–7.72 (m, 2H), 7.54 (s, 1H), 7.43–7.20 (m, 3H), 7.20–7.06 (m, 3H), 6.91 (s, 1H), 4.33–4.29 (m, 2H). ^13^C-NMR (101 MHz, DMSO-*d_6_*) δ 169.19, 165.24, 162.10, 161.12, 145.62, 140.49, 140.20, 133.86, 130.98, 129.56, 128.33, 128.31, 127.56, 127.53, 127.53, 126.12, 123.82, 120.08, 119.10, 37.07. ESI-MS: (*m*/*z*) calculated for C_23_H_18_ClN_3_O_3_S: 455.05; found: 456 (M + H)^+^; analysis calculated (%): C, 61.13; H, 4.01; Cl, 7.84; N, 9.30; O, 10.62; S, 7.09; found: C, 60.12; H, 2.01; Cl, 9.84; N, 9.32; O, 11.62; S, 6.09.

***4-(((7-chloro-3-(4-chlorophenyl)-4-oxo-3,4-dihydroquinazolin-2-yl)thio)methyl)-N-hydroxybenzamide (5n):***

Off-white solid; yield 64%, m.p. 230–235 °C. ^1^H-NMR (400 MHz, DMSO-*d_6_*) δ 7.98 (s, 1H), 7.78–7.72 (m, 2H), 7.60 (s, 1H), 7.37 (s, 1H), 7.35–7.30 (m, 2H), 7.29–7.19 (m, 4H), 7.00 (s, 1H), 4.40 (s, 2H), ^13^C-NMR (100 MHz, DMSO-*d_6_*) δ 168.19, 163.21, 158.16, 142.62, 140.49, 140.20, 137.44, 133.86, 130.68, 130.76, 130.76, 130.01, 130.01, 129.56, 129.56, 128.31, 127.53, 127.53, 123.82, 120.14, 118.10, 36.07. ESI-MS: (*m*/*z*) calculated for C_22_H_15_C_l2_N_3_O_3_S: 471.34; found: 472 (M + H)^+^; analysis calculated (%): C, 55.94; H, 3.20; Cl, 15.01; N, 8.90; O, 10.16; S, 6.79; found: C, 55.90; H, 3.24; Cl, 15.02; N, 8.93; O, 10.12; S, 5.79.

***4-(((7-chloro-3-(3,4-dimethylphenyl)-4-oxo-3,4-dihydroquinazolin-2-yl)thio)methyl)-N-hydroxybenzamide (5o):***

White solid; yield 60%, m.p. 223–230 °C. ^1^H-NMR (400 MHz, DMSO-*d_6_*) δ 8.65 (bs, 1H), 8.12–8.00 (m, 1H), 7.91–7.79 (m, 2H), 7.78–7.56 (m, 2H), 7.55–7.38 (m, 2H), 7.37–7.03 (m, 3H), 4.64 (s, 2H), 2.27 (s, 3H) 2.28 (s, 3H). ^13^C-NMR (100 MHz, DMSO-*d_6_*) δ 167.19, 163.21, 158.16, 140.62, 140.49, 140.20, 139.20, 137.11, 136.85, 133.86, 132.32, 129.58, 129.56, 129.56, 128.31, 127.67, 127.53, 127.53, 124.82, 120.14, 118.10, 37.07, 22.37, 19.45. ESI-MS: (*m*/*z*) calculated for C_24_H_20_ClN_3_O_3_S: 465.09; found: 466 (M + H)^+^; analysis calculated (%): C, 61.87; H, 4.33; Cl, 7.61; N, 9.02; O, 10.30; S, 6.88; found: C, 60.87; H, 4.32; Cl, 7.59; N, 9.02; O, 10.12; S, 6.25.

***N-hydroxy-4-(((5-methyl-4-oxo-3-phenyl-3,4-dihydroquinazolin-2-yl)thio)methyl)benzamide (5p):***

Off-white solid; yield 63%, m.p. 182–187 °C. ^1^H-NMR (400 MHz, DMSO-*d_6_*) δ 7.84–7.69 (m, 2H), 7.33 (dd, *J* = 16.3, 9.3 Hz, 8H), 7.16 (d, *J* = 1.3 Hz, 2H), 6.82 (s, 1H), 4.33–4.29 (m, 2H), 2.56–2.52 (m, 3H). ^13^C-NMR (101 MHz, DMSO-*d_6_*) δ 162.81, 161.42, 156.09, 146.19, 139.23, 136.26, 135.72, 134.62, 129.85, 128.83, 127.09, 126.15, 124.71, 119.82, 36.26, 17.83. ESI-MS: (*m*/*z*) calculated for C_23_H_19_N_3_O_3_S: 417.11; found: 418 (M + H)^+^; analysis calculated (%): C, 66.17; H, 4.59; N, 10.07; O, 11.50; S, 7.68; found: C, 66.15; H, 4.55; N, 10.02; O, 11.55; S, 7.62.

***4-(((3-(2-fluorophenyl)-5-methyl-4-oxo-3,4-dihydroquinazolin-2-yl)thio)methyl)-N-hydroxybenzamide (5q):***

White solid; yield 65%, m.p. 185–190 °C. ^1^H-NMR (400 MHz, DMSO-*d_6_*) δ 7.85–7.71 (m, 2H), 7.45–7.26 (m, 2H), 7.17 (d, *J* = 10.0 Hz, 2H), 7.10 (d, *J* = 9 Hz, 2H), 6.89 (s, 1H), 4.38–4.34 (m, 2H), 2.58–2.54 (m, 3H). ^13^C-NMR (101 MHz, DMSO-*d_6_*) δ 162.85, 160.94, 151.29, 145.99, 136.36, 134.87, 131.84, 130.72, 129.27, 127.38, 125.64, 125.14, 121.84, 119.39, 114.52, 36.09, 17.96. ESI-MS: (*m*/*z*) calculated for C_23_H_18_FN_3_O_3_S: 435.11; found: 436 (M + H)^+^; analysis calculated (%): C, 63.40; H, 4.12; F, 4.35; N, 9.64; O, 11.03; S, 7.35.

***N-hydroxy-4-(((5-methyl-4-oxo-3-(o-tolyl)-3,4-dihydroquinazolin-2-yl)thio)methyl)benzamide (5r):***

Off-white solid; yield 64%, m.p. 163–170 °C. ^1^H-NMR (400 MHz, DMSO-*d_6_*) δ 9.29–8.43 (bs, 1H), 7.92 (dd, *J* = 10.5, 7.7 Hz, 1H), 7.79–7.59 (m, 2H), 7.55–7.42 (m, 4H), 7.42–7.26 (m, 4H), 4.50 (s, 2H), 2.57 (s, 3H), 2.02 (s, 3H). ^13^C-NMR (101 MHz, DMSO-*d_6_*) δ 164.30, 161.05, 156.00, 146.18, 140.48, 136.60, 135.93, 135.48, 134.74, 132.81, 131.53, 130.73, 129.89, 129.17, 127.83, 127.33, 124.77, 119.66, 35.87, 17.86, 17.39. ESI-MS: (*m*/*z*) calculated for C_24_H_21_N_3_O_3_S: 431.13 11; found: 432 (M + H)^+^; analysis calculated (%): C, 66.80; H, 4.91; N, 9.74; O, 11.12; S, 7.43; found: C, 66.82; H, 4.90; N, 9.75; O, 11.10; S, 5.43.

***4-(((3-(4-chlorophenyl)-5-methyl-4-oxo-3,4-dihydroquinazolin-2-yl)thio)methyl)-N-hydroxybenzamide (5s):***

Off-white solid; yield 62%, m.p. 156–160 °C. ^1^H-NMR (400 MHz, DMSO-*d_6_*) δ 7.95–7.85 (m, 1H), 7.73–7.67 (m, 2H), 7.67–7.62 (m, 2H), 7.61–7.57 (m, 2H), 7.55–7.51 (m, 2H), 7.40–7.33 (m, 2H), 4.86 (s, 2H), 2.87–2.22 (s, 3H). ^13^C-NMR (101 MHz, DMSO-*d_6_*) δ 164.40, 161.78, 155.83, 145.81, 139.25, 135.84, 135.24, 135.11, 134.65, 133.59, 131.88, 131.49, 130.08, 129.12, 127.12, 126.13, 124.71, 119.86, 36.28, 17.63. ESI-MS: (*m*/*z*) calculated for C_23_H_18_ClN_3_O_3_S: 451.08; found: 452 (M + H)^+^; analysis calculated (%): C, 61.10; H, 4.05; Cl, 7.82; N, 9.33; O, 10.32; S, 7.05; found: C, 61.09; H, 4.06; Cl, 7.82; N, 9.32; O, 10.34; S, 7.03.

## 4. Conclusions

Using novel quinazolin-4-(3H)-ones as the capping groups and benzhydroxamic acid as the linker and metal-binding group, a series of novel quinazoline-4-(3H)-one derivatives were designed and synthesized as histone deacetylase 6 (HDAC6) inhibitors. The majority of the compounds showed sub micromolar activities with distinct preference for HDAC6. Intracellular target engagement of the final compounds was demonstrated by a dose-dependent increase in acetylated α-tubulin with a limited effect on acetyl-H3, indicating a preference for the HDAC6 isozyme. Some of these compounds also showed promising antiproliferative activity in HCT116, MCF7, and B16 tumor cell lines and the HEK293 cell line. Moreover, **5c**—the most active compound with an IC_50_ of 13.65 μM in the MCF7 cell line—caused cell-cycle arrest in the G2 phase, promoted apoptosis, and showed a significant reduction in colony formation of tumor cells. In summary, we presented a novel class of selective HDAC6 inhibitors with promising antitumor properties. The compounds possess remarkable biochemical and intracellular selectivity for HDAC6 enabling the reduction in off-target effects in further optimization cycles. Consequently, the presented class of compounds may serve as a useful chemical starting point to develop drug candidates with an improved safety profile against tumor diseases.

## Figures and Tables

**Table 1:** Synthesized compounds with physicochemical properties.

Entry	R^1^	R^2^	Physical Status	m.p (°C)	% Yield
**5a**	H	4-Cl	off white	164–166	66
**5b**	H	H	off white	158–160	52
**5c**	H	4-CH_3_	white	141–142	65
**5d**	H	4-CH_2_CH_3_	off white	155–157	64
**5e**	H	4-Br	pale yellow	173–175	58
**5f**	H	3.4-CH_3_	white	142–144	63
**5g**	H	3-CH_3_	off white	143–144	65
**5h**	H	3-Cl	off white	138–140	70
**5i**	H	2-F	white	134–136	54
**5j**	7-Cl	H	pale yellow	235–236	62
**5k**	7-Cl	2-CH_3_	white	178–180	63
**5l**	7-Cl	4-CH_3_	white	173–175	59
**5m**	7-Cl	2-F	white	155–156	60
**5n**	7-Cl	4-Cl	off white	234–235	64
**5o**	7-Cl	3.4-CH_3_	white	229–230	60
**5p**	5-CH_3_	H	off white	185–187	63
**5q**	5-CH_3_	2-F	white	189–190	65
**5r**	5-CH_3_	2-CH_3_	white	169–170	64
**5s**	5-CH_3_	4-Cl	off white	158–160	62

**Table 2:** IC_50_ values against class I HDAC8, class IIa HDAC4, and class IIb HDAC6.

Compound	IC_50_ ± SD (nM) *		
	HDAC4	HDAC6	HDAC8
**5a**	4500 ± 1200	5600 ± 600	24,000 ± 1000
**5b**	2300 ± 1100	150 ± 10	1400 ± 300
**5c**	720 ± 210	580 ± 50	2300 ± 500
**5d**	640 ± 370	280 ± 60	2300 ± 800
**5e**	1100 ± 400	1100 ± 100	3000 ± 500
**5f**	1600 ± 900	490 ± 120	3000 ± 1000
**5g**	870 ± 310	260 ± 20	790 ± 160
**5h**	1400 ± 500	170 ± 30	1100 ± 300
**5i**	8400 ± 1200	360 ± 40	2900 ± 600
**5j**	4300 ± 1200	650 ± 70	4000 ± 1000
**5k**	>35,000	410 ± 150	1300 ± 100
**5l**	19,000 ± 1000	3900 ± 400	17,000 ± 1000
**5m**	560 ± 240	210 ± 50	3300 ± 900
**5n**	6700 ± 1100	320 ± 30	140 ± 20
**5o**	7100 ± 1100	390 ± 220	14,000 ± 1000
**5p**	400 ± 140	220 ± 50	2700 ± 600
**5q**	6100 ± 1200	320 ± 20	2700 ± 300
**5r**	1700 ± 700	540 ± 70	3900 ± 800
**5s**	910 ± 330	860 ± 60	5900 ± 800
**SAHA**	27,000	86	5300
**Trichostatin A**	3300	17	360
**Tubastatin**	4700 ± 1500	160 ± 30	2700 ± 1800

* IC_50_ values are shown as the mean ± SD. The assay was carried out in triplicate.

**Table 3:** MTT assay showing the cytotoxic activity of final compounds on human cancer cell lines.

Compound	IC_50_ ± SD (µM) *			
	HCT116	MCF7	B16	HEK 293
**5a**	46.24 ± 20	26.48 ± 3.50	48.82 ± 9.68	30.94 ± 226.74
**5b**	17.62 ± 1.77	24.31 ± 5.97	28.88 ± 2.32	26.41 ± 05
**5c**	12.38 ± 1.41	13.65 ± 0.83	15 ± 3.34	35.07 ± 18.72
**5d**	34.52 ± 10.45	21.69 ± 2.47	29.83 ± 8.72	>50
**5e**	46.4 ± 17.79	44.63 ± 1.07	26.057 ± 1.27	>50
**5f**	>50	>50	>50	>50
**5g**	30.67 ± 15.75	25.34 ± 3.21	23.01 ± 13.66	>50
**5h**	>50	>50	>50	>50
**5i**	40.39 ± 16.02	>50	>50	>50
**5j**	>50	>50	>50	>50
**5k**	>50	>50	>50	>50
**5l**	>50	>50	>50	>50
**5m**	>50	>50	>50	>50
**5n**	>50	>50	33.91 ± 5.12	>50
**5o**	>50	>50	>50	>50
**5p**	>50	>50	>50	>50
**5q**	>50	>50	>50	>50
**5r**	>50	>50	>50	>50
trip	>50	>50	>50	>50
**SAHA**	7.99 ± 1.68	44.41 ± 8.30	32.88 ± 18.04	11.92 ± 3.42
**Trichostatin A**	18.31 ± 5.63	42.48 ± 11.33	28.87 ± 13.01	15.29 ± 1.45
**Tubastatin**	1.34 ± 0.04	12.65 ± 4.68	1.99 ± 0.25	1.26 ± 0.29
**Ricolinostat**	40.46 ± 7.69	>50	89.23 ± 12.51	66.78 ± 10.57
**Erlotinib**	>50	>50	>50	>50

* IC_50_ values are shown as the mean ± SD. The assay was carried out in triplicate.

## Data Availability

Data is contained within the article or supplementary material.

## Supplementary Materials

The following supporting information can be downloaded at: https://www.mdpi.com/article/10.3390/ijms241311044/s1 [61].
