# Supplementary material for: Design, Synthesis, and Biological Evaluation of Novel Quinazolin-4(3H)-One-Based Histone Deacetylase 6 (HDAC6) Inhibitors for Anticancer Activity
Source: Int J Mol Sci. 2023 Jul 3;24(13):11044. doi: 10.3390/ijms241311044 (PMC10341573; doi:10.3390/ijms241311044)
Supplement: Supplementary file 1 [file ijms-24-11044-s001.zip › ijms-2439471-supplementary.pdf]

Design, Synthesis and Biological Evaluation of Novel Quinazolin-4(3H)-one Based Histone Deacetylase 6 (HDAC6) Inhibitors for Anticancer Activity

Yogesh Mahadu Khetmalis[a], Ashna Fathima[b], Markus Schweipert[c], Cécile Debarnot[c], Naga Venkata Madhusudhan Rao Bandaru[a], Sankaranarayanan Murugesan[d], Trinath Jamma[b], Franz-Josef Meyer-Almes*[c], Kondapalli Venkata Gowri Chandra Sekhar*[a]

[a]*Department of Chemistry, Birla Institute of Technology & Science-Pilani, Hyderabad campus, Hyderabad-500078, Telangana, India.*

[b]*Department of Biological Sciences, Birla Institute of Technology & Science-Pilani, Hyderabad campus, Hyderabad-500078, Telangana, India.*

[c]*Department of Chemical Engineering and Biotechnology, University of Applied Sciences Darmstadt, Haardtring 100, 64295 Darmstadt, Germany.*

[d]*Medicinal Chemistry Research Laboratory, Department of Pharmacy, Birla Institute of Technology and Science Pilani, Pilani Campus, Pilani-333031, Rajasthan. India.*

---

*Corresponding authors

E-mail: kvgc@hyderabad.bits-pilani.ac.in (KVGCS); franz-josef.meyer-almes@h-da.de (FJMA)

Content:

Figure S1. Shown is the residual enzyme activity of HDAC4, 6 and 8. in the presence of 35 µM compounds measured in duplicate (Table S1). A residual activity of 1 means no inhibition.

Figure S2: Redocking of ligand into the crystal structure of HDAC4, HDAC6 and HDAC8

Figure S3: Overlay of energy-minimized docking poses of compounds 5a-c in HDAC4, HDAC6 and HDAC8

Table S1. List of every residual activity, which are shown in Figure S1.

Table S2. *In silico* predicted physico-chemical parameters of the titled compounds

Table S3. Docking scores (GBVI/WSA dG) and IC_{50}-values against HDAC4, 6 and 8

Chemical analytics:

^{1}H NMR spectras of final compounds (5a-5s)

^{13}C NMR spectras of final compounds (5a-5s):

Mass spectras of final compounds (5a-5s):

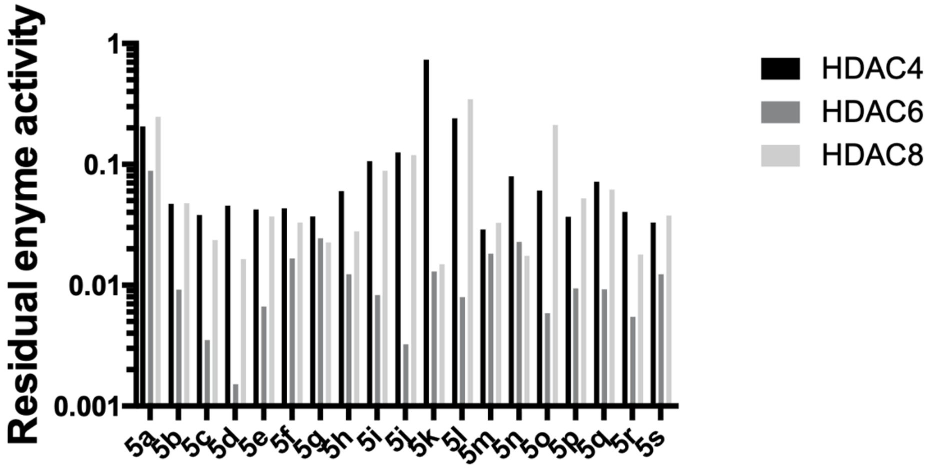

Figure S1. Shown is the residual enzyme activity of HDAC4, 6 and 8. in the presence of 35 μM compounds measured in duplicate **(Table S1).** A residual activity of 1 means no inhibition.

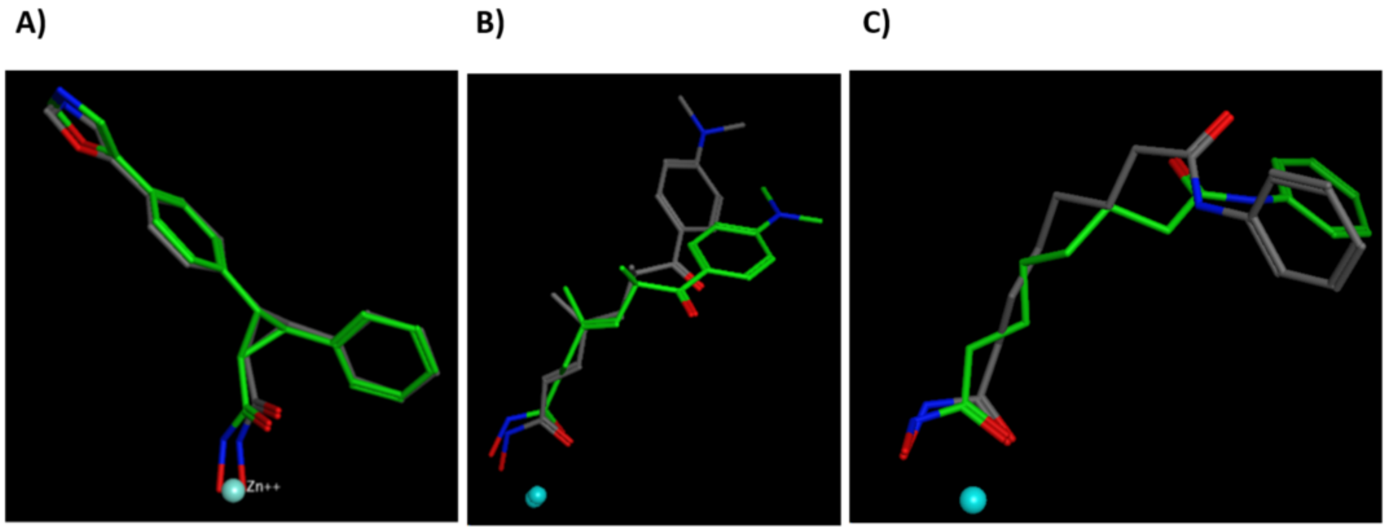

Figure S2. (A) Redocking of ligand into the crystal structure of the binding pocket of HDAC4 (PDB-ID: 4CBY) shows perfect overlap between the docked ligand (gray) and the X-ray binding pose (green). **(B)** Redocking into HDAC6 (PDB-ID: 5EDU) shows very good agreement in the lower part of the binding pocket, but considerable deviation between the head group, which protrude freely into solution. C) Redocking of ligand into the crystal structure of the binding pocket of HDAC8 (PDB-ID: 1T69) shows very good overlap between the docked ligand (grey) and the X-ray binding pose (green) in the lower part of the binding pocket. The phenyl head group, which protrudes into free solution, shows more deviation.

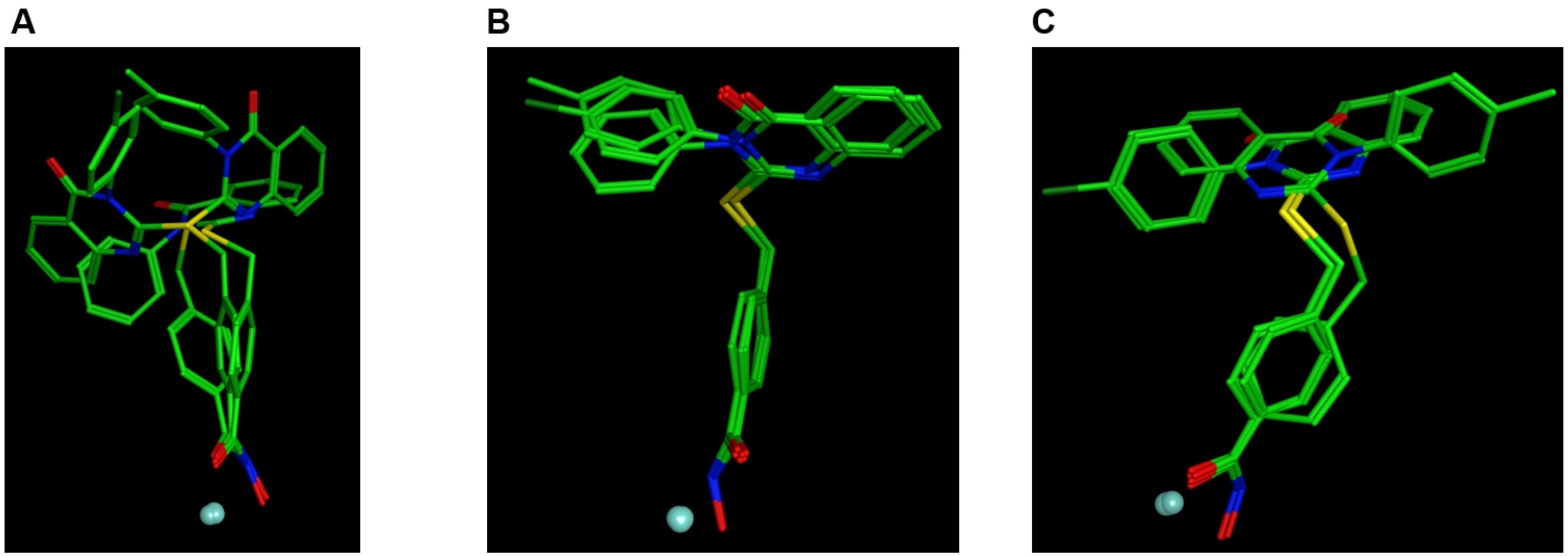

Figure S3. Overlay of energy-minimized docking poses of compounds 5a-c within the binding pockets of (A) HDAC4 (PDB-ID: 4CBY), (B) HDAC6 (PDB-ID: 5EDU) and (C) HDAC8 (PDB-ID: 1T69). Cyan beads represent the catalytic zinc ions.

Table S1. List of every residual activity, which are shown in **Figure S2.**

**Compound**	**HDAC4**		**HDAC6**		**HDAC8**	
**5a**	0.194404	0.216831	0.077992	0.098287	0.215139	0.28015
**5b**	0.045894	0.048698	0.013369	0.005031	0.045445	0.049903
**5c**	0.040223	0.036002	0.003059	0.003964	0.026078	0.021322
**5d**	0.046217	0.044928	0.000409	0.002628	0.01771	0.015115
**5e**	0.04467	0.03974	0.003189	0.010169	0.038257	0.035911
**5f**	0.042576	0.043961	0.010072	0.023193	0.03638	0.029747
**5g**	0.0293	0.04467	0.016277	0.03263	0.020597	0.024671
**5h**	0.055078	0.064905	0.019315	0.005343	0.027337	0.028617
**5i**	0.100961	0.111852	0.013261	0.003372	0.088274	0.088338
**5j**	0.131894	0.119843	0.004901	0.001594	0.122059	0.116172

**5k**	0.742141	0.730283	0.014392	0.011548	0.0168	0.013068
**5l**	0.261329	0.218989	0.008963	0.006991	0.3384	0.351155
**5m**	0.02669	0.031105	0.021545	0.015049	0.030323	0.035229
**5n**	0.080501	0.078535	0.019336	0.026349	0.017696	0.017333
**50**	0.054369	0.067515	0.004083	0.007638	0.221218	0.203344
**5p**	0.044122	0.029526	0.010805	0.007993	0.053614	0.050969
**5q**	0.073186	0.07077	0.007993	0.010514	0.063874	0.059778
**5r**	0.038645	0.041963	0.005989	0.004998	0.018101	0.017675
**5s**	0.035422	0.030621	0.00739	0.017279	0.04359	0.031965

Table S2. *In silico* predicted physico-chemical parameters of the titled compounds

Entry	mol MW	Donor HB	Accpt HB	Log Po/w	Rule of Five	TPSA	ESOL Class	GI absorption
**5a**	437.9	2	4	2.96	0	109.52	Moderately soluble	High
**5b**	403.45	2	4	3.04	0	109.52	Moderately soluble	High
**5c**	417.48	2	4	2.87	0	109.52	Moderately soluble	High
**5d**	431.51	2	4	3.26	0	109.52	Moderately soluble	High
**5e**	482.35	2	4	3.01	0	109.52	Moderately soluble	High
**5f**	431.51	2	4	2.7	0	109.52	Moderately soluble	High
**5g**	417.48	2	4	3.34	0	109.52	Moderately soluble	High
**5h**	437.9	2	4	3.41	0	109.52	Moderately soluble	High
**5i**	421.44	2	5	3.05	0	109.52	Moderately soluble	High
**5j**	437.9	2	4	3.43	0	109.52	Moderately soluble	High
**5k**	451.93	2	4	3.25	0	109.52	Moderately soluble	High
**5l**	451.93	2	4	3.12	0	109.52	Moderately soluble	High
**5m**	455.89	2	5	3.19	0	109.52	Moderately soluble	High
**5n**	472.34	2	4	2.52	0	109.52	Moderately soluble	High
**5o**	465.95	2	4	3.21	0	109.52	Poorly soluble	High
**5p**	417.48	2	4	2.79	0	109.52	Moderately soluble	High
**5q**	435.47	2	5	3.25	0	109.52	Moderately soluble	High
**5r**	431.51	2	4	2.56	0	109.52	Moderately soluble	High
**5s**	451.93	2	4	2.68	0	109.52	Moderately soluble	High

Description: Lipinski rule of five – Number of violations of Lipinski's rule of five. The rules are: mol MW - 130.0 – 725.0, log Po/w - –2.0 – 6.5, donor HB ≤ 5, accept HB ≤10 and maximum 4 violations. **Log Po/w**:Log of partition coefficient; **TPSA**: Topological polar surface area:TPSA < 140 is essential for good absorption; **ESOL:** Estimated aqueous solubility in mg/mL; **GI:** Gastrointestinal.

Table S3. Docking scores (GBVI/WSA dG) and IC_{50}-values against HDAC4, 6 and 8. The PDB-Id's used for docking are indicated in brackets.

Compound	HDAC4 (PDB:4CBY)	HDAC6 (PDB:5EDU)	HDAC8 (PDB:1T69)	HDAC4 IC_{50} (nM)	HDAC6 IC_{50} (nM)	HDAC8 IC_{50} (nM)
**5a**	-11.5	-13.1	-12.0	4500	5600	24000
**5b**	-11.5	-13.1	-12.1	2300	150	1400
**5c**	-11.6	-13.1	-12.0	720	580	2300
**Cpd 31 (4CBY)**	-13.2	-	-	20*	-	360*
**Trichostatin A (5EDU)**	-	-13.7	-	3300	17	360
**SAHA (1T69)**	-	-	-12.0	27000	86	5300
**Tubastatin**	-	-	-	4700	160	2700

*Values are from Bürli et al's work [1]

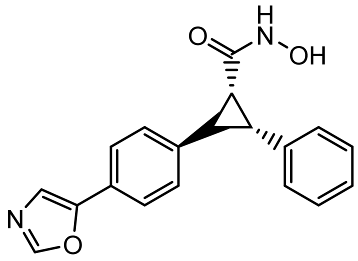

Structure of **Cpd 31**

1. Bürli, R. W., Luckhurst, C. A., Aziz, O., Matthews, K. L., Yates, D., Lyons, K. A., Dominguez, C. (2013). Design, synthesis, and biological evaluation of potent and selective class IIa histone deacetylase (HDAC) inhibitors as a potential therapy for Huntington's disease. *Journal of Medicinal Chemistry*, *56*(24), 9934-9954.

^{1}H NMR spectras of final compounds (5a-5s):

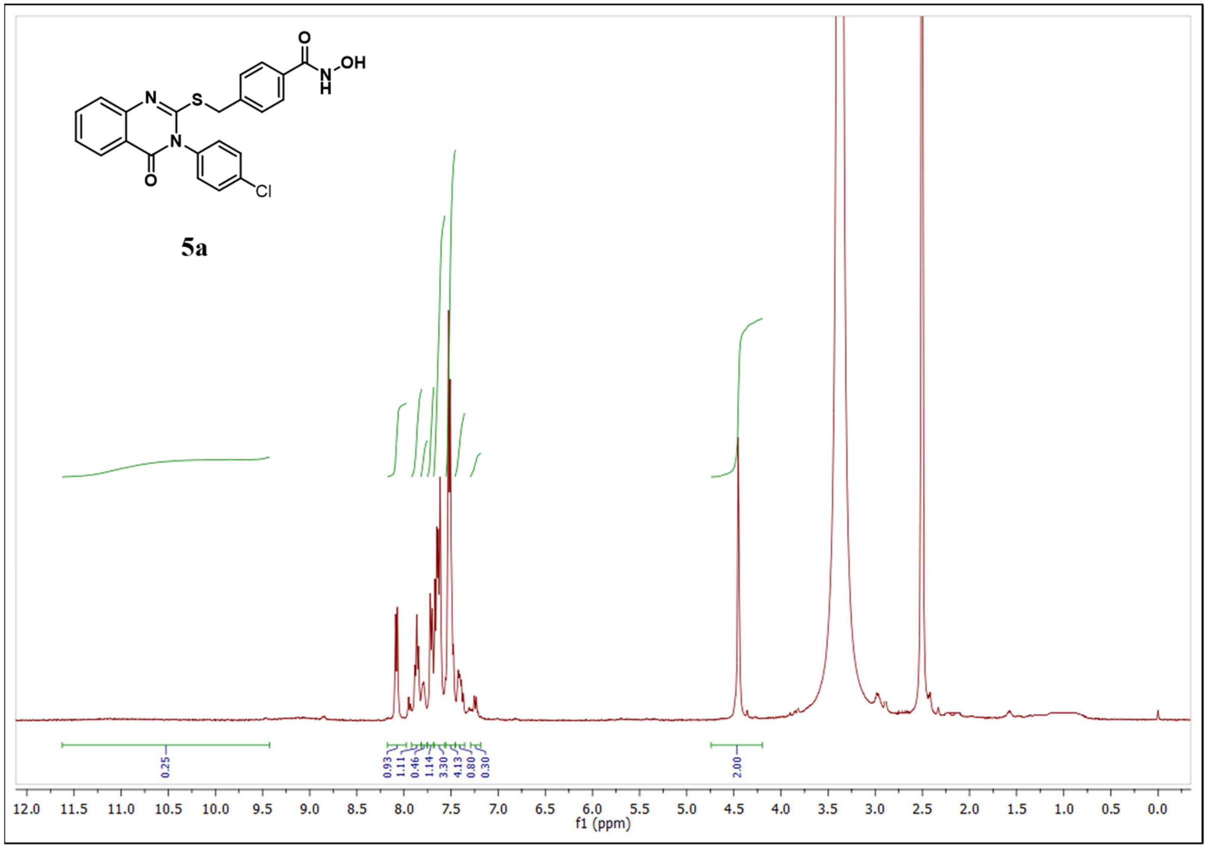

^{1}H NMR spectrum of compound **5a (**DMSO-d_6 400 MHz)

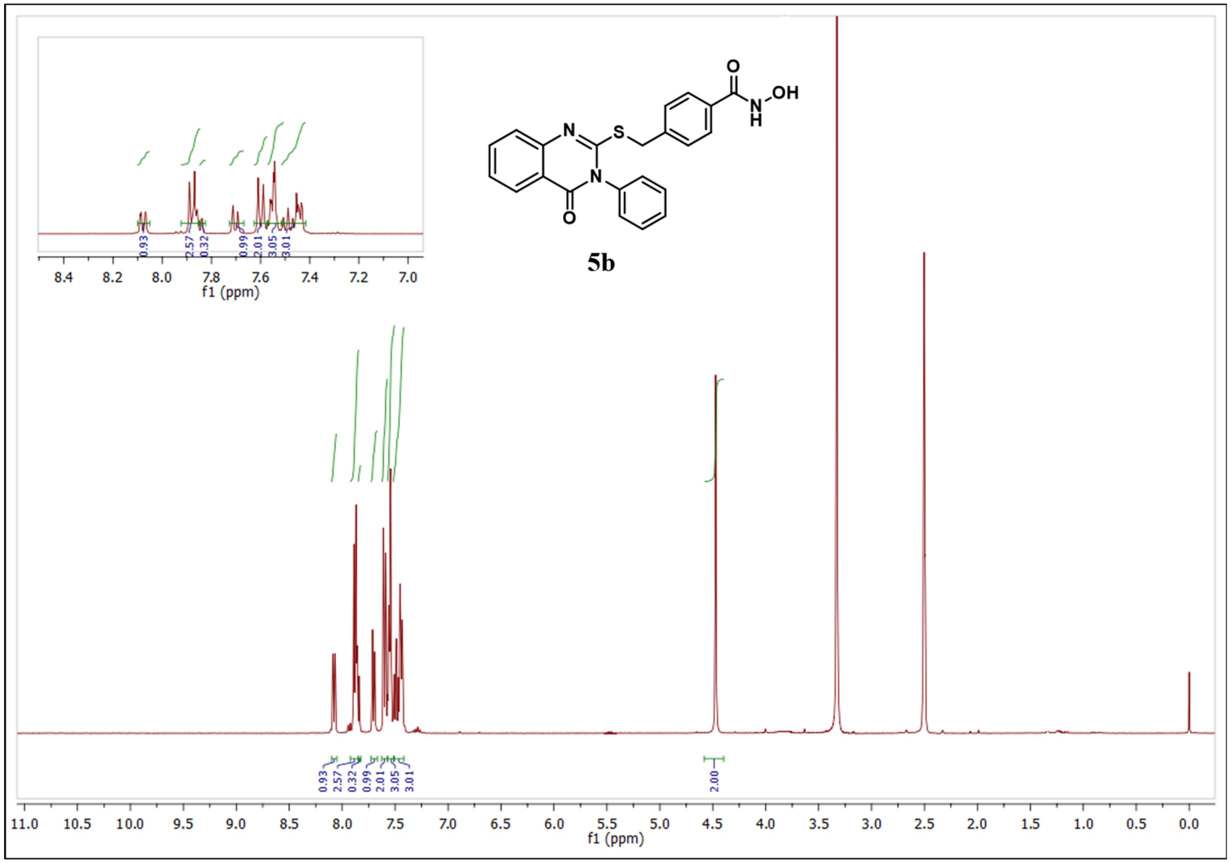

^{1}H NMR spectrum of compound **5b** **(**DMSO-d_6 400 MHz)

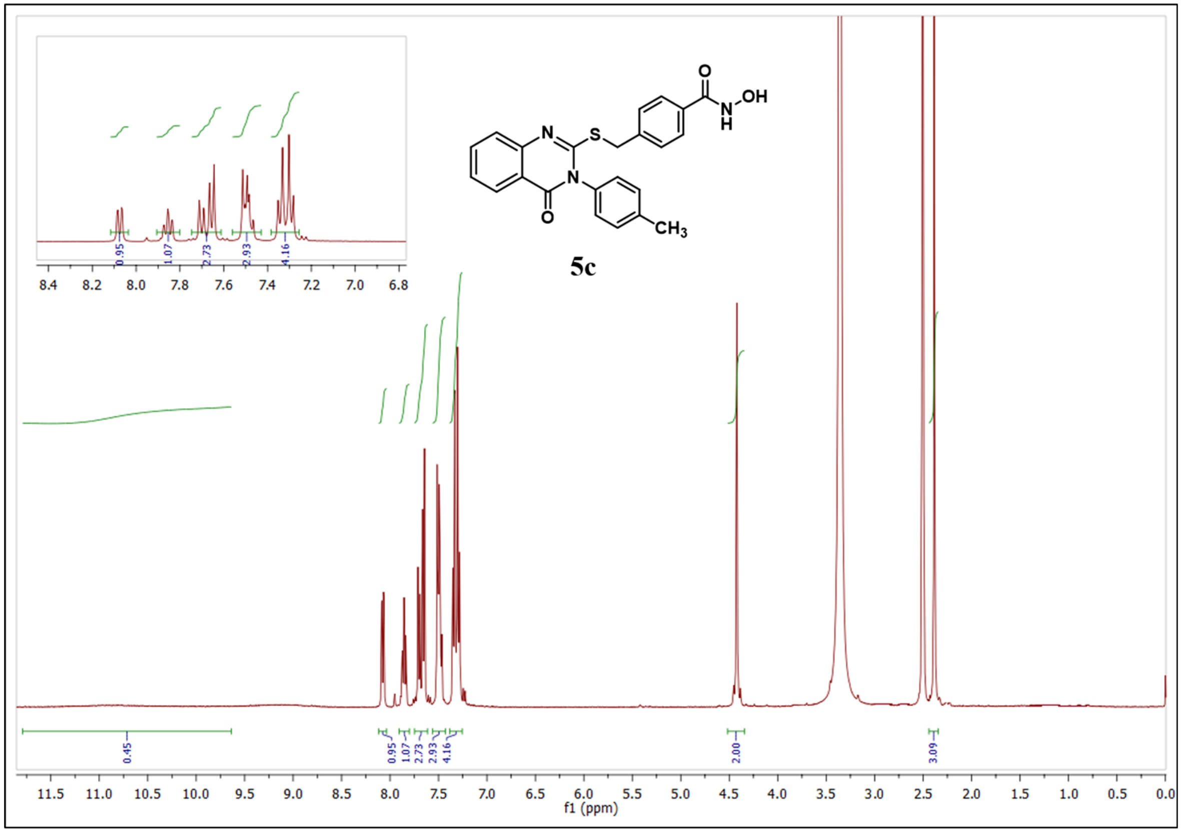

[1]**H NMR** spectrum of compound **5c (**DMSO-d_6 400 MHz)

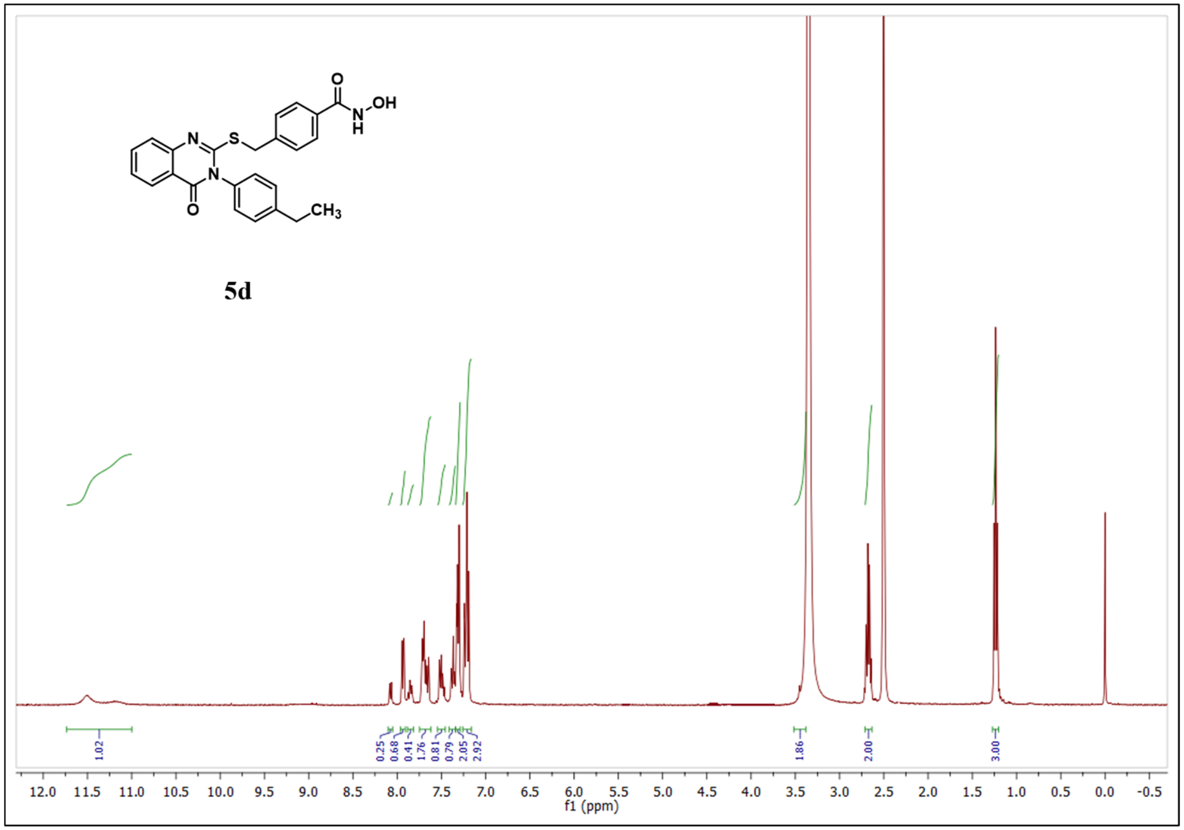

[1]**H NMR** spectrum of compound **5d (**DMSO-*d₆* 400 MHz)

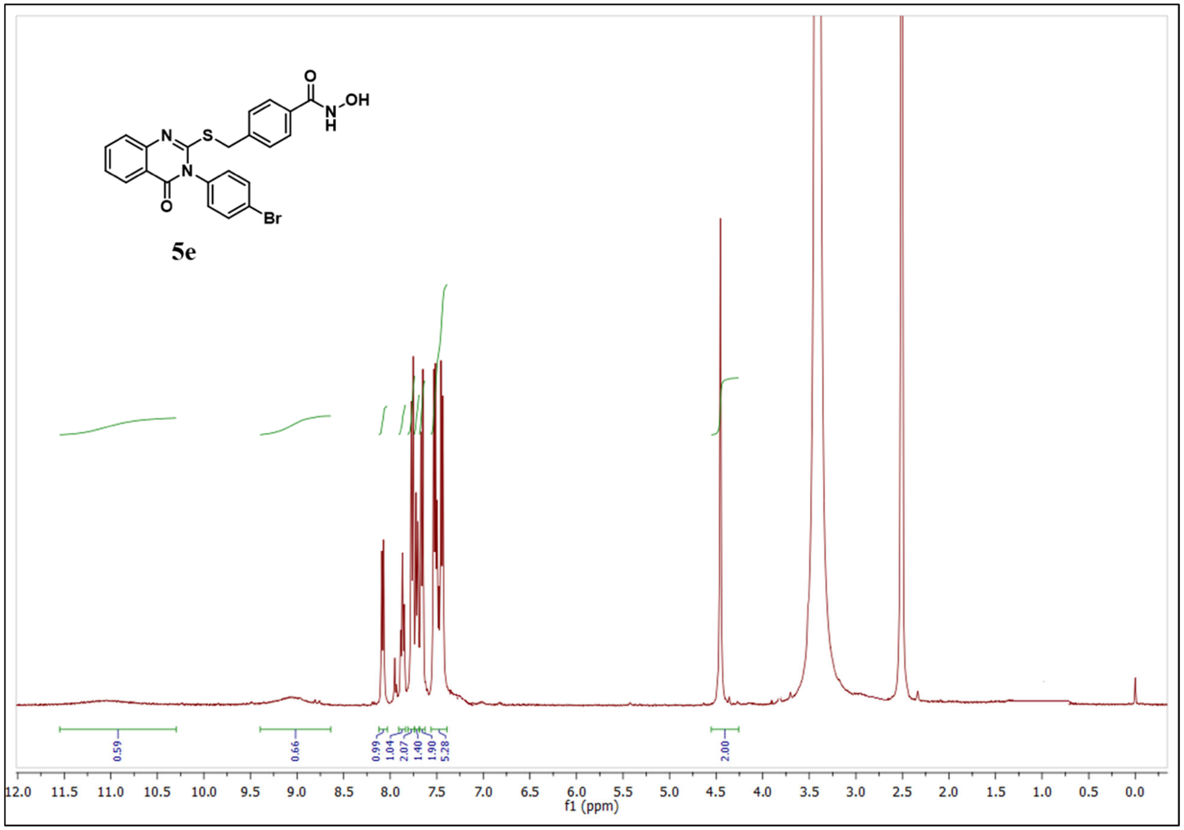

[1]**H NMR** spectrum of compound **5e (**DMSO-*d*$_6$ 400 MHz)

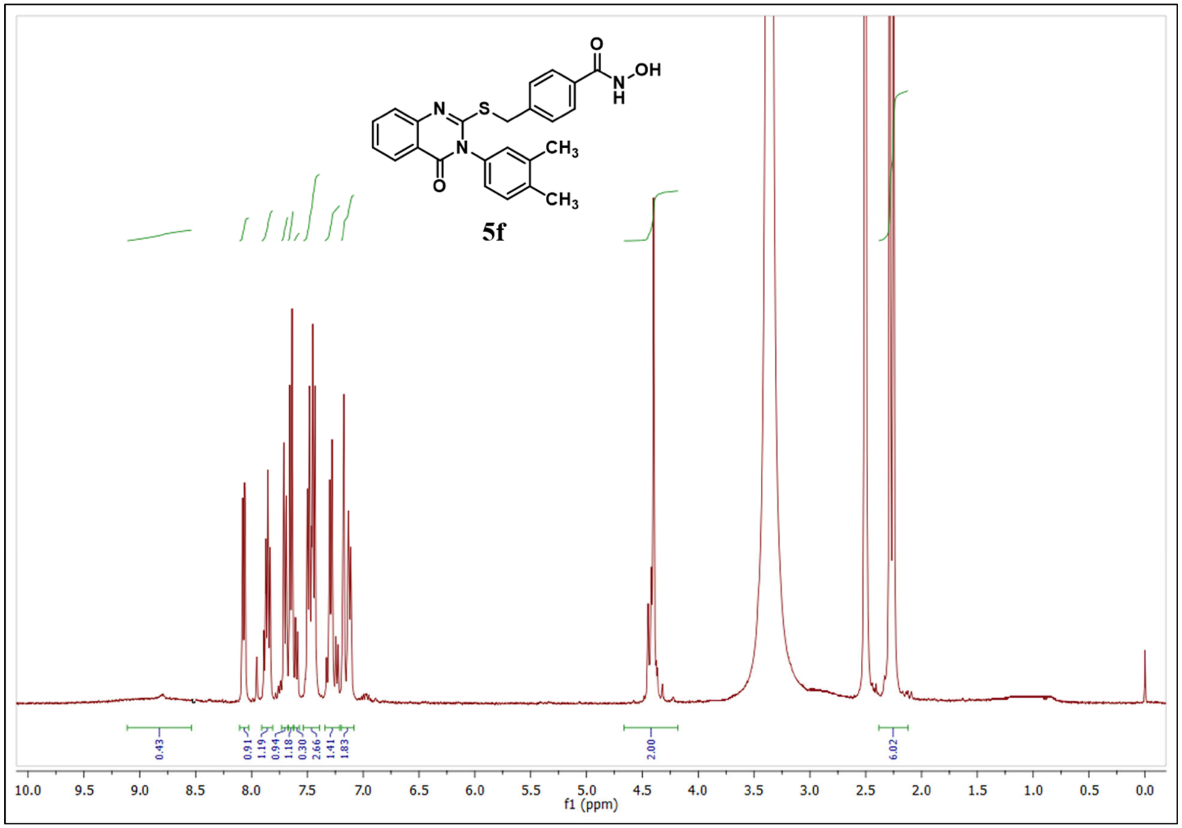

^{1}H NMR spectrum of compound **5f (**DMSO-d_6 400 MHz)

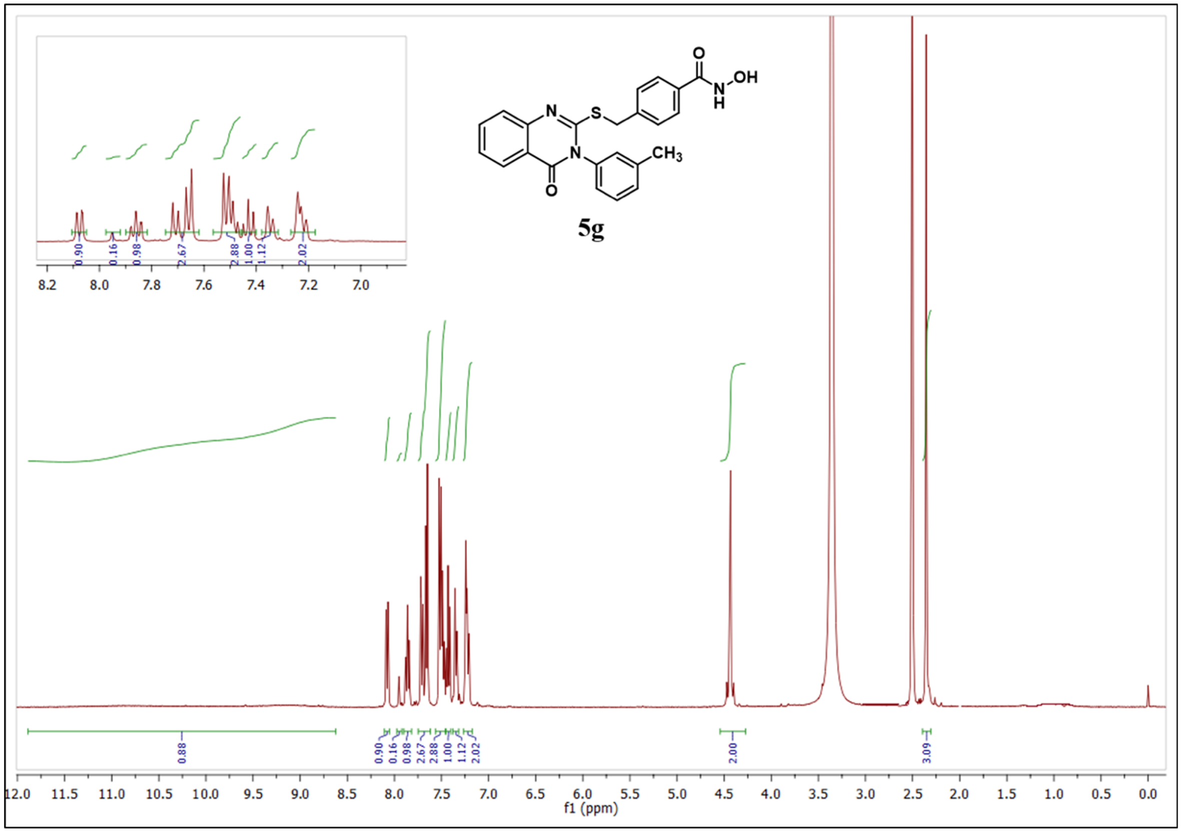

^{1}H NMR spectrum of compound **5g (**DMSO-d_6 400 MHz)

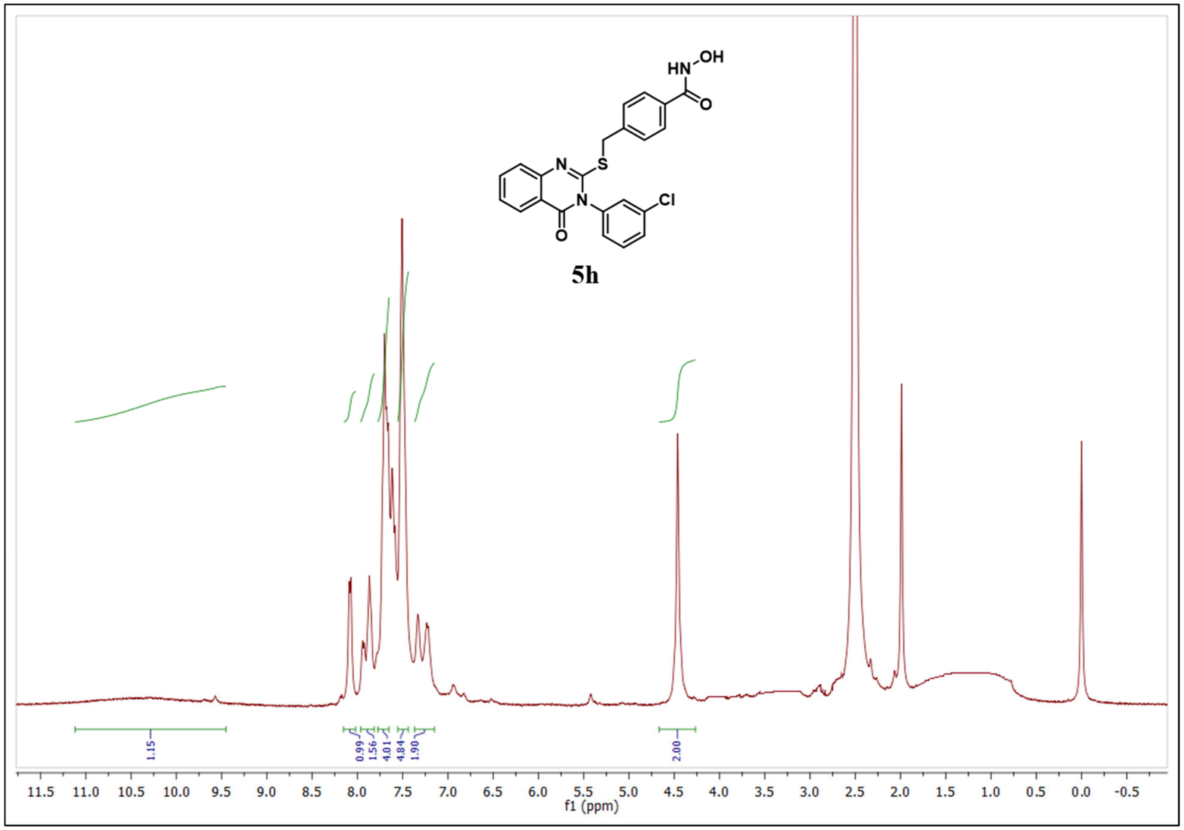

[1]**H NMR** spectrum of compound **5h (**DMSO-*d*₆ 400 MHz)

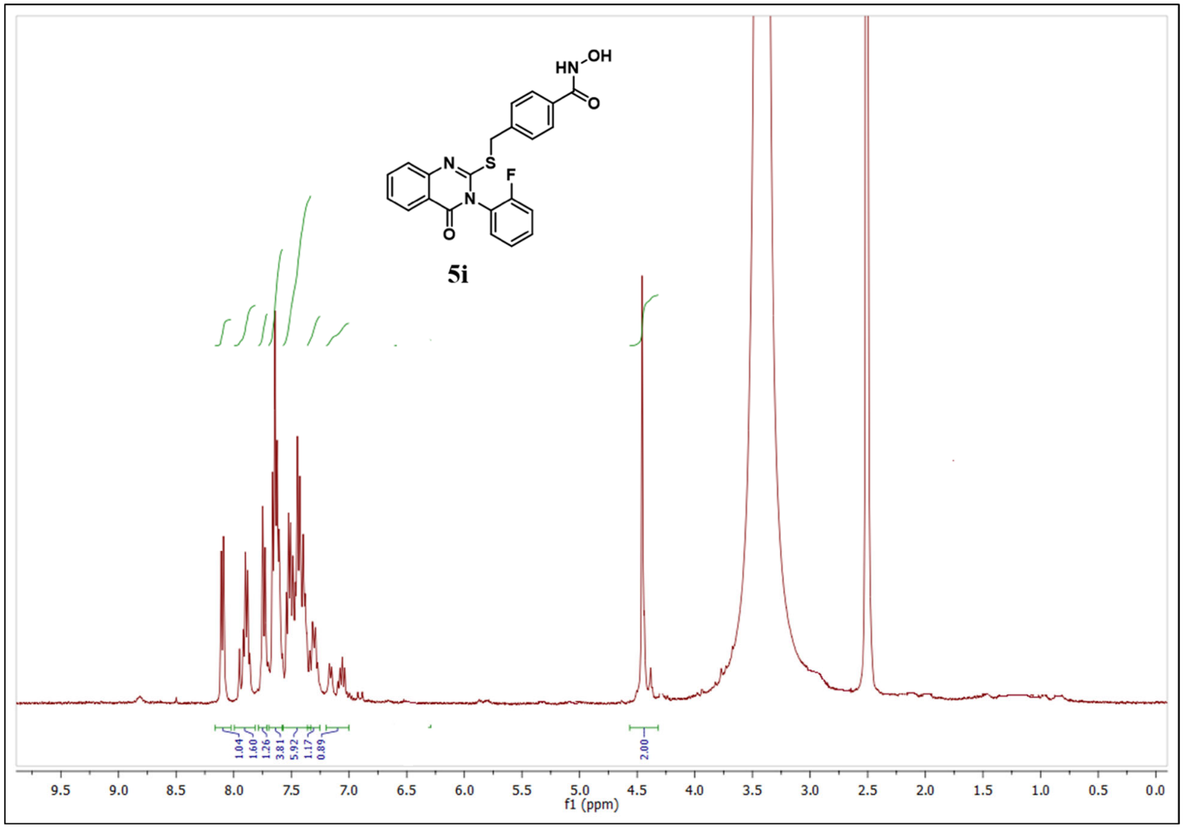

^{1}H NMR spectrum of compound **5i (**DMSO-d_6 400 MHz)

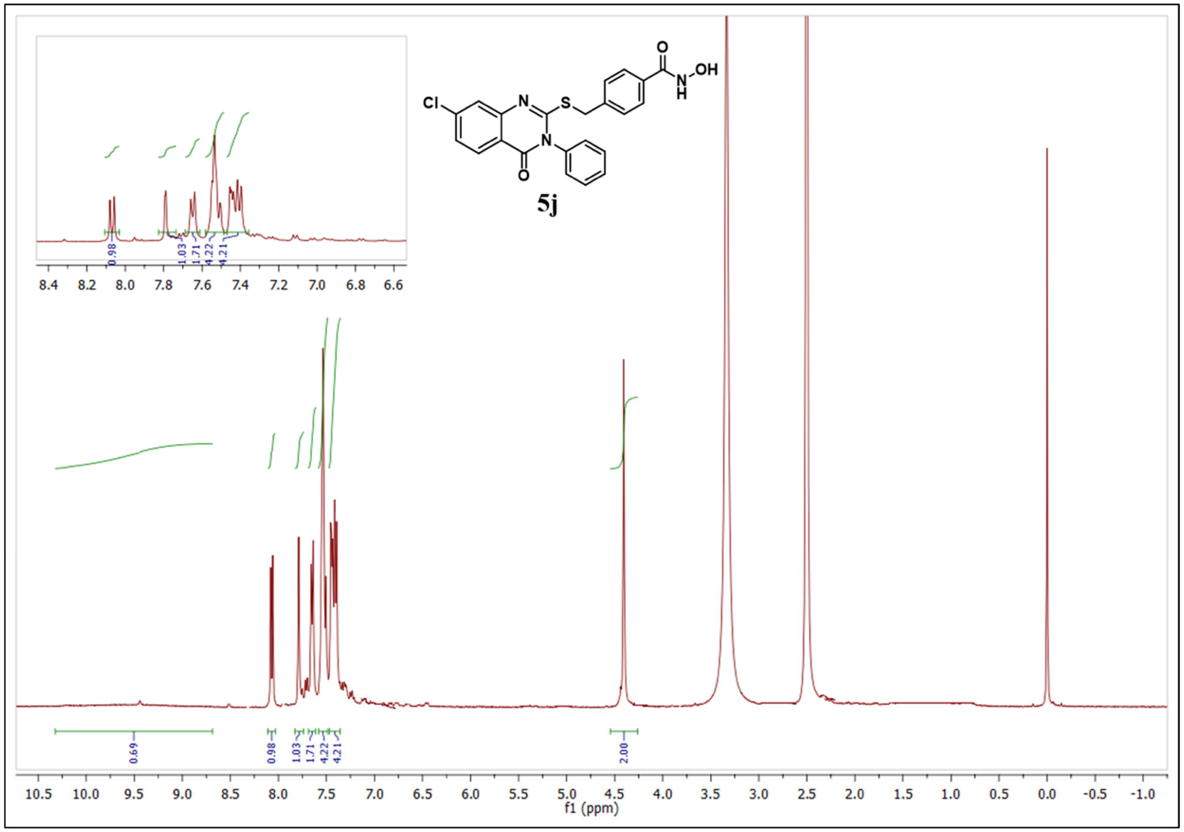

^{1}H NMR spectrum of compound **5j (**DMSO-d_6 400 MHz)

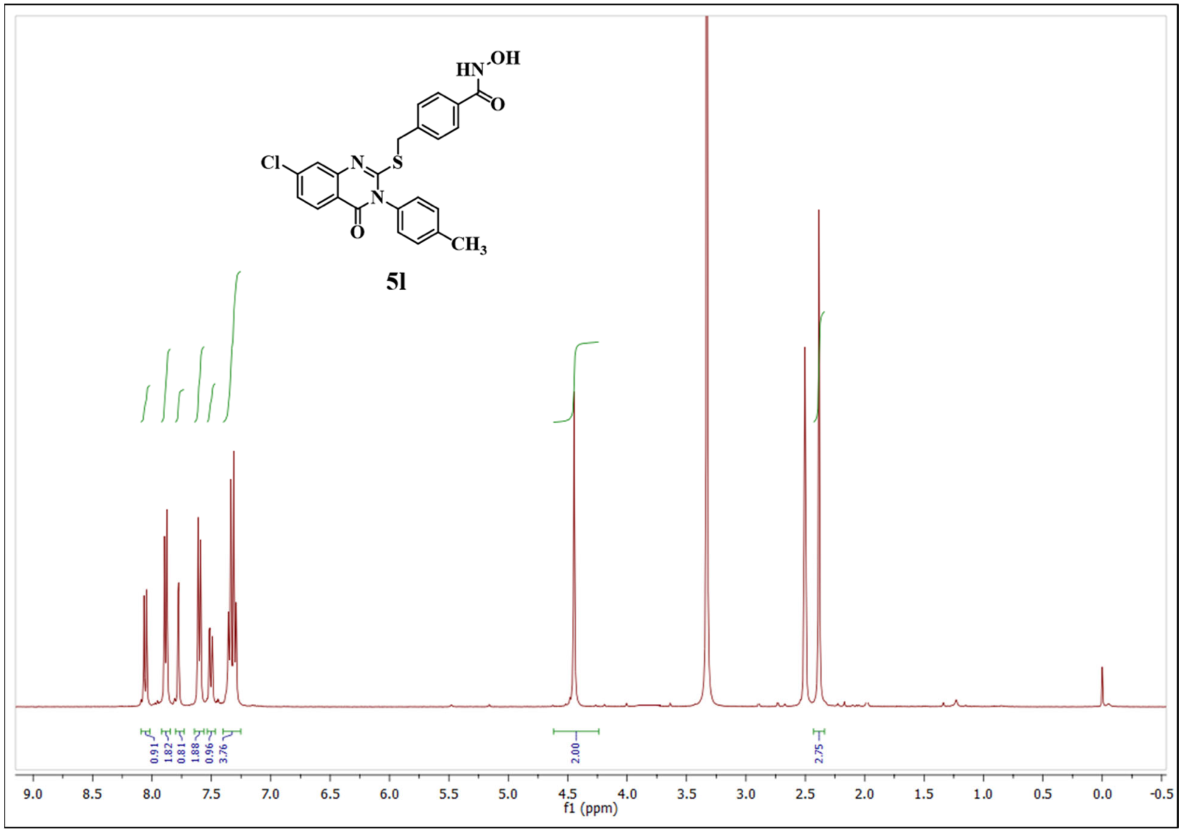

^{1}H NMR spectrum of compound **5l (**DMSO-*d₆* 400 MHz)

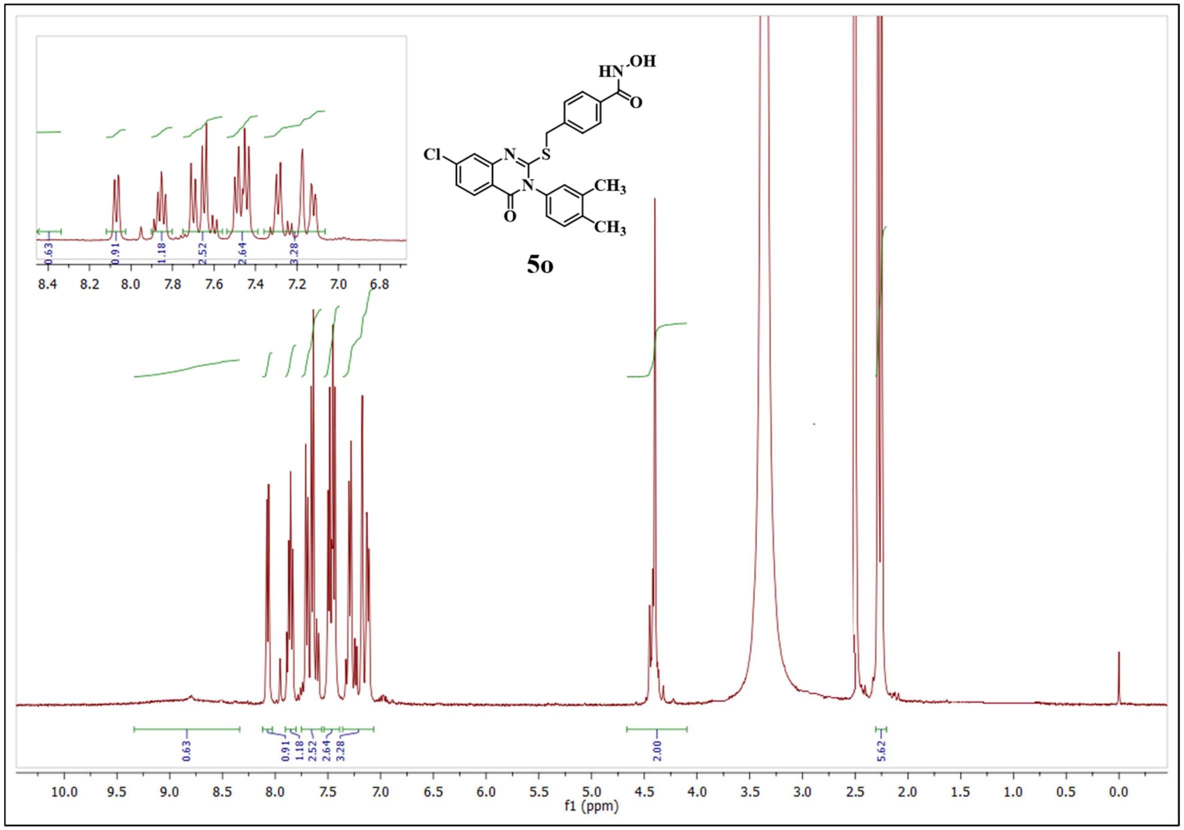

[1]**H NMR** spectrum of compound **5o (**DMSO-d_6 400 MHz)

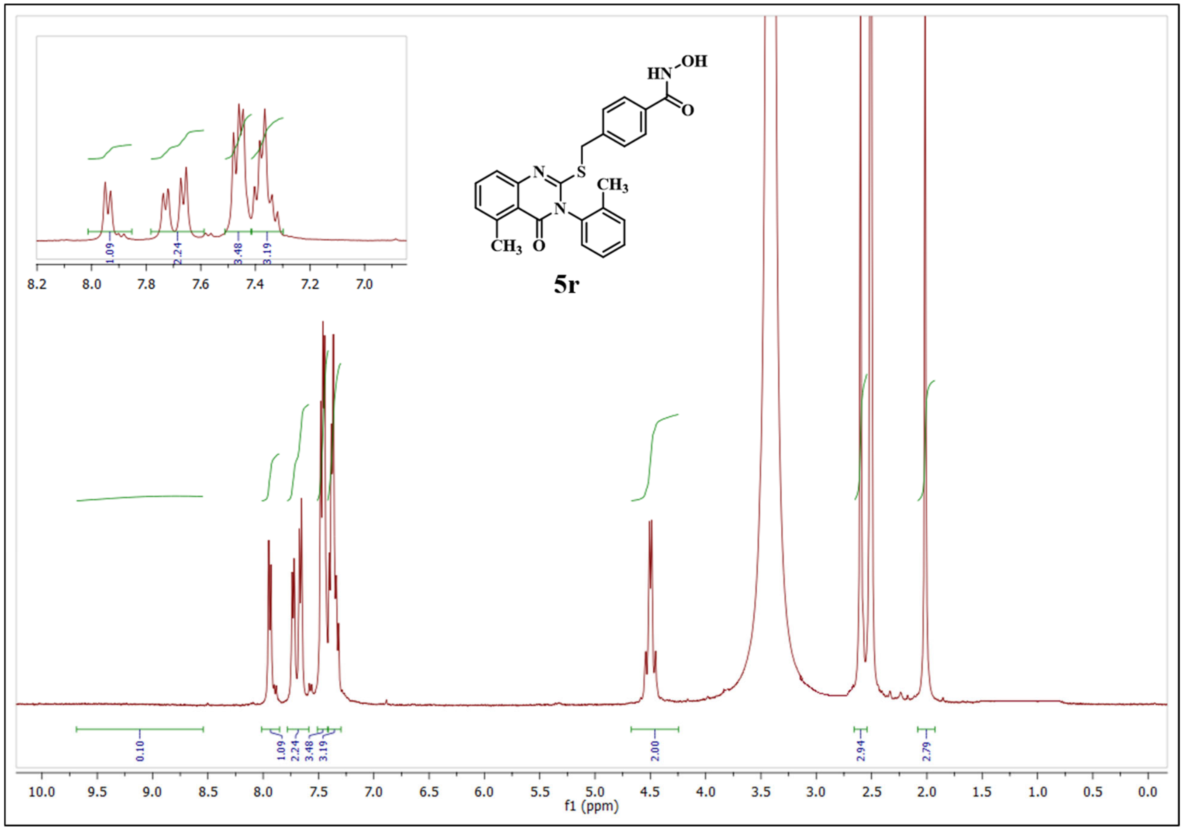

^{1}**H NMR** spectrum of compound **5r** (DMSO-d_6 400 MHz)

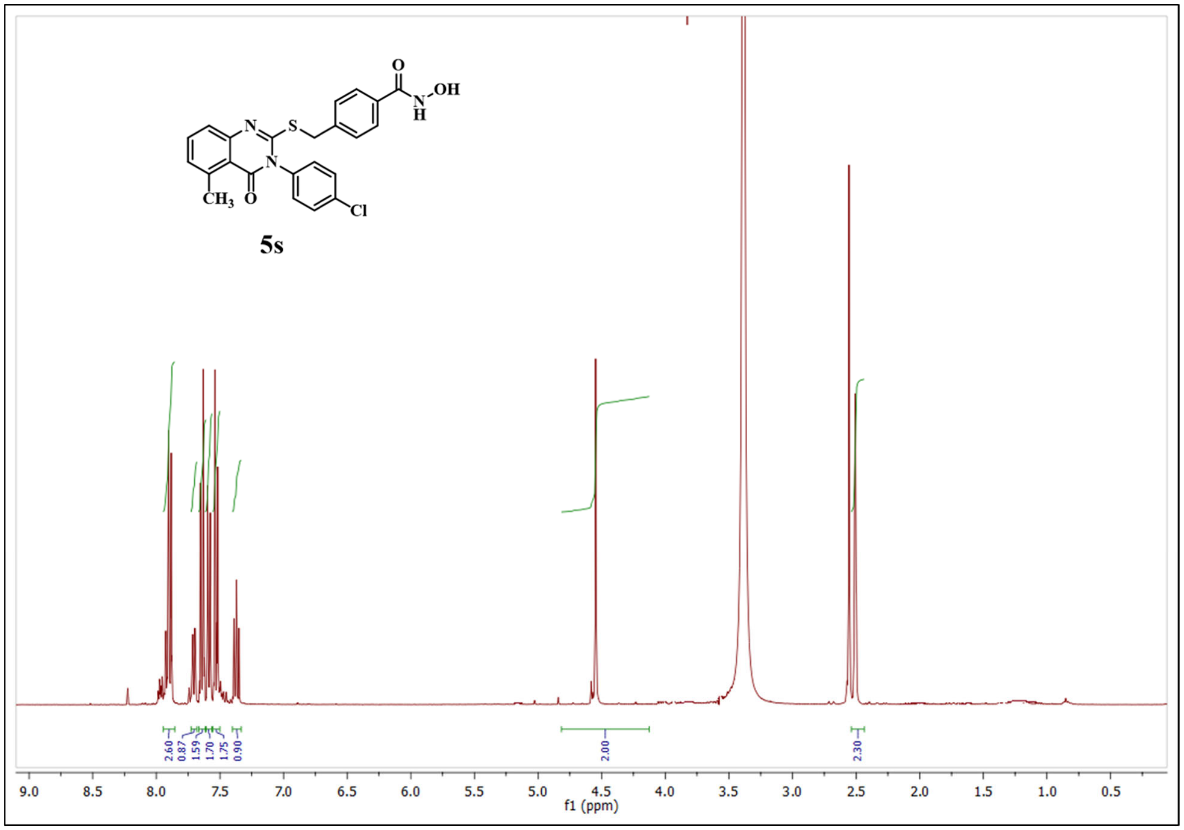

[1]**H NMR** spectrum of compound **5s (**DMSO-d_6 400 MHz)

^{13}C NMR spectras of final compounds (5a-5s):

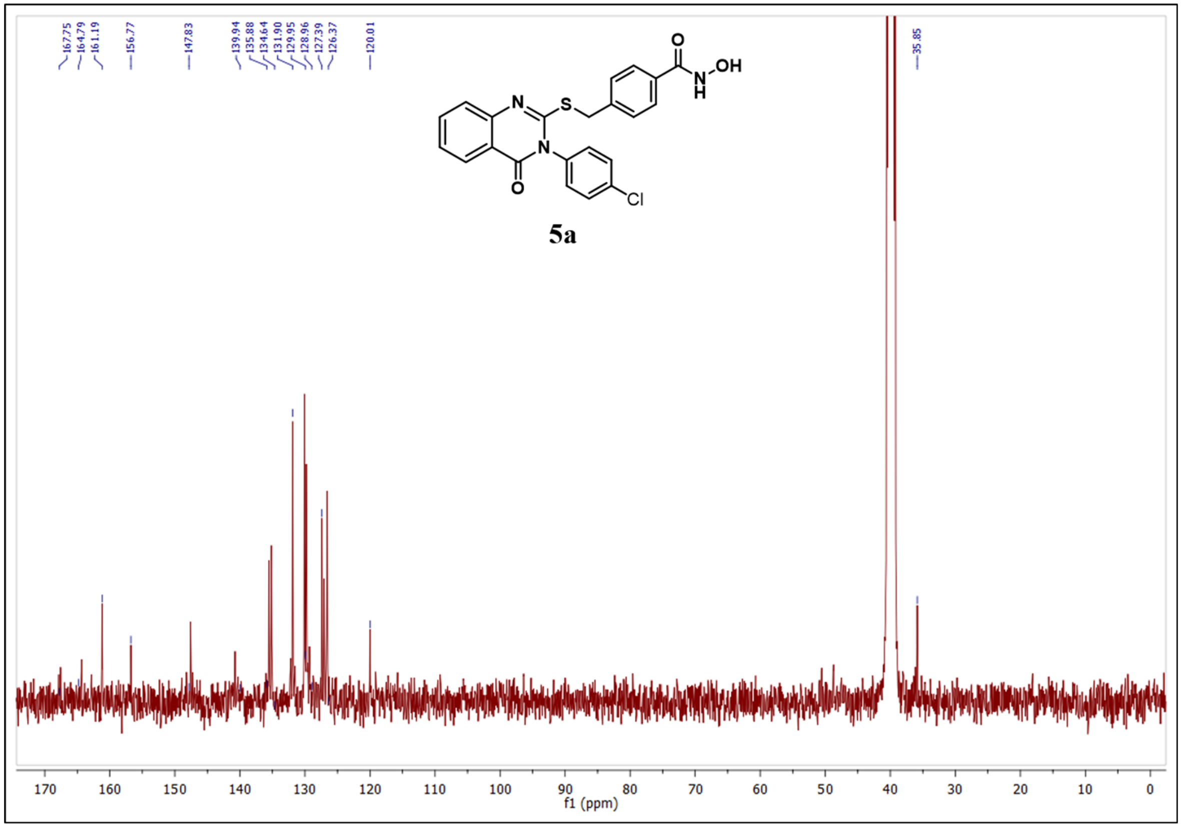

^{13}**C NMR** spectrum of compound **5a** (DMSO-d_6 101 MHz)

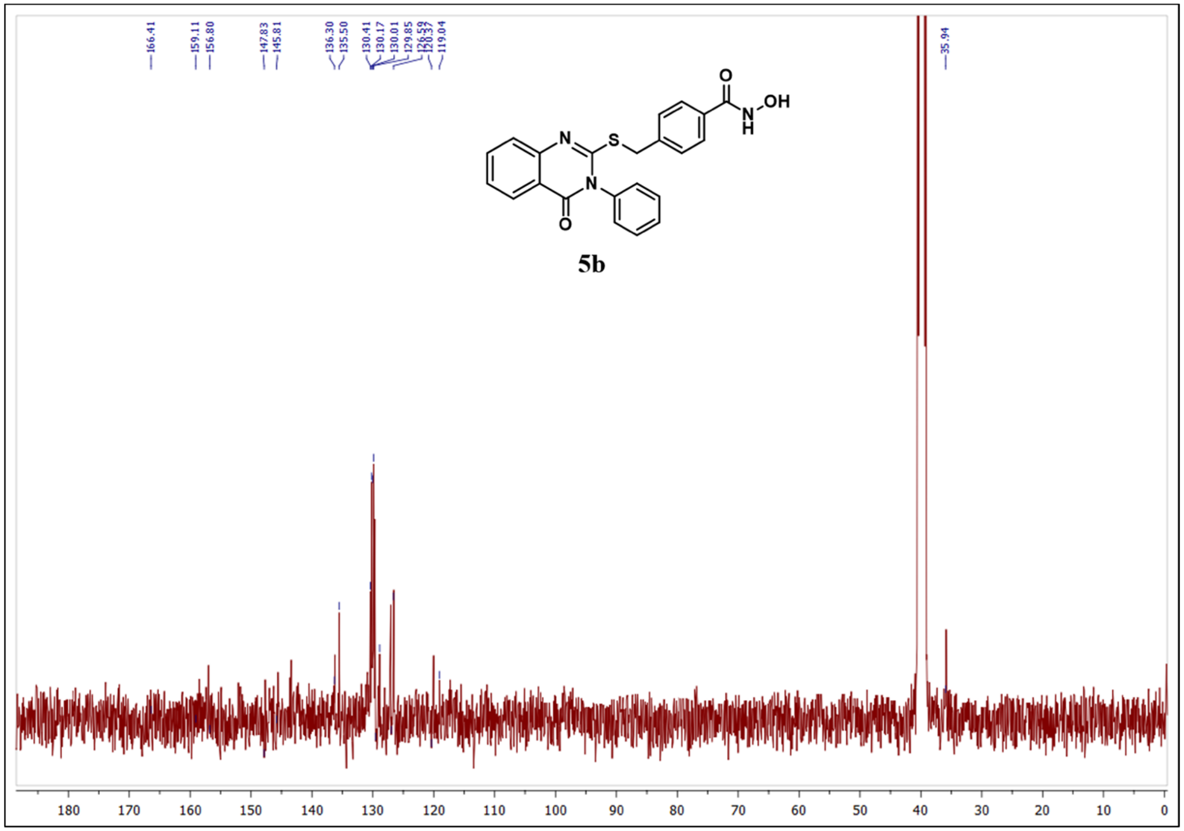

^{13}C NMR spectrum of compound **5b** (DMSO-d_6 101 MHz)

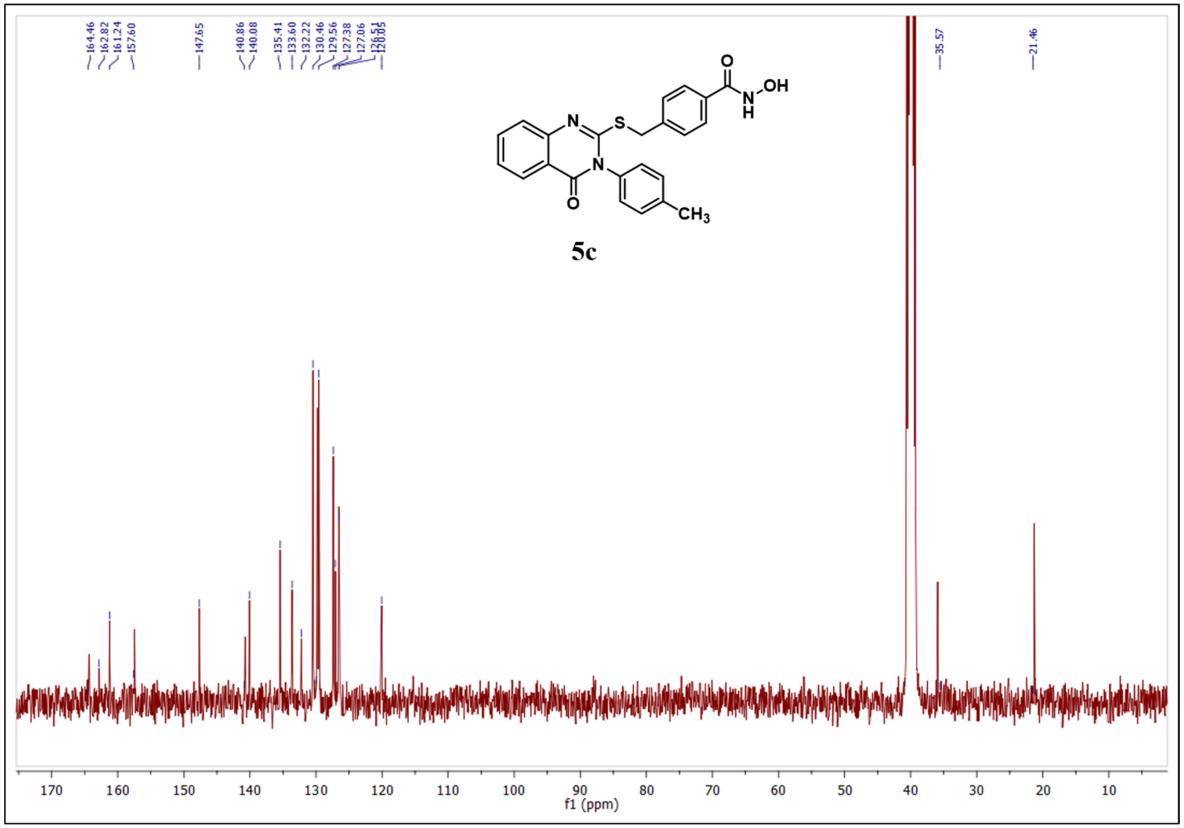

^{13}C NMR spectrum of compound **5c** (DMSO-d_6 101 MHz)

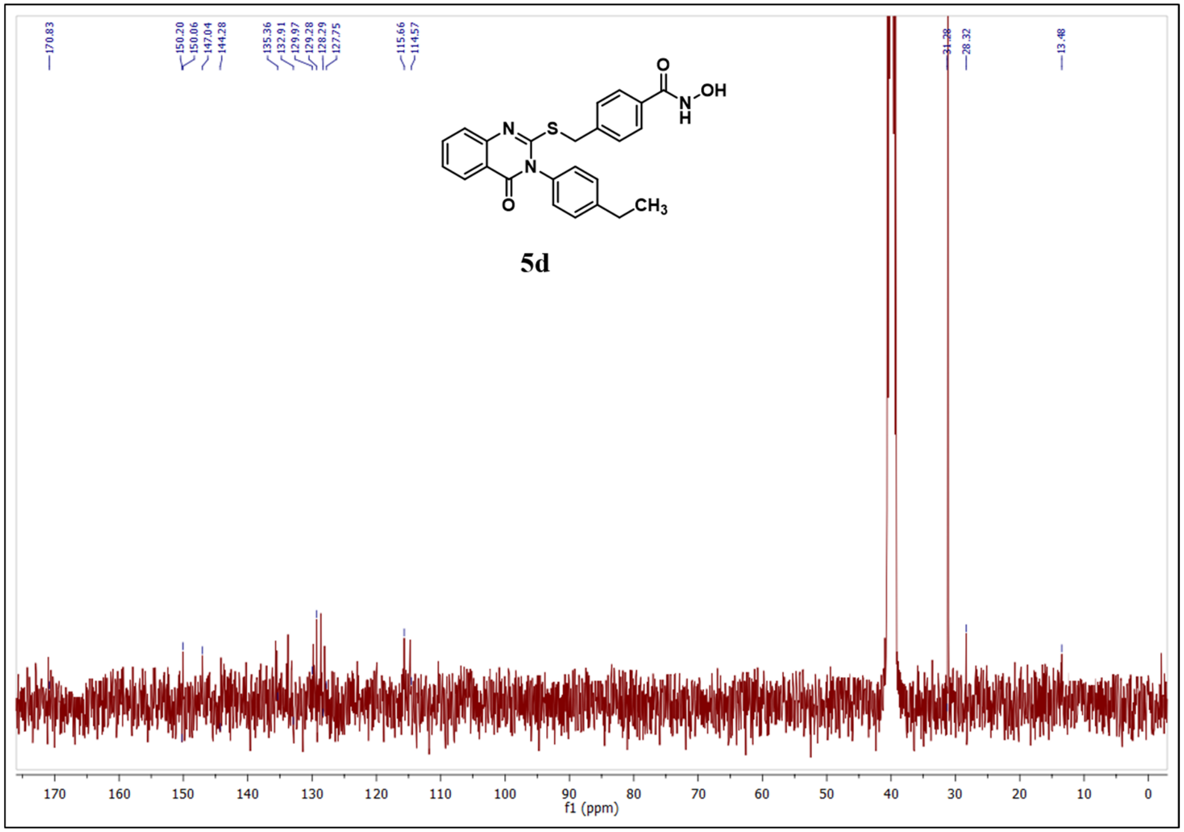

^{13}C **NMR** spectrum of compound **5d** (DMSO-d_6 101 MHz)

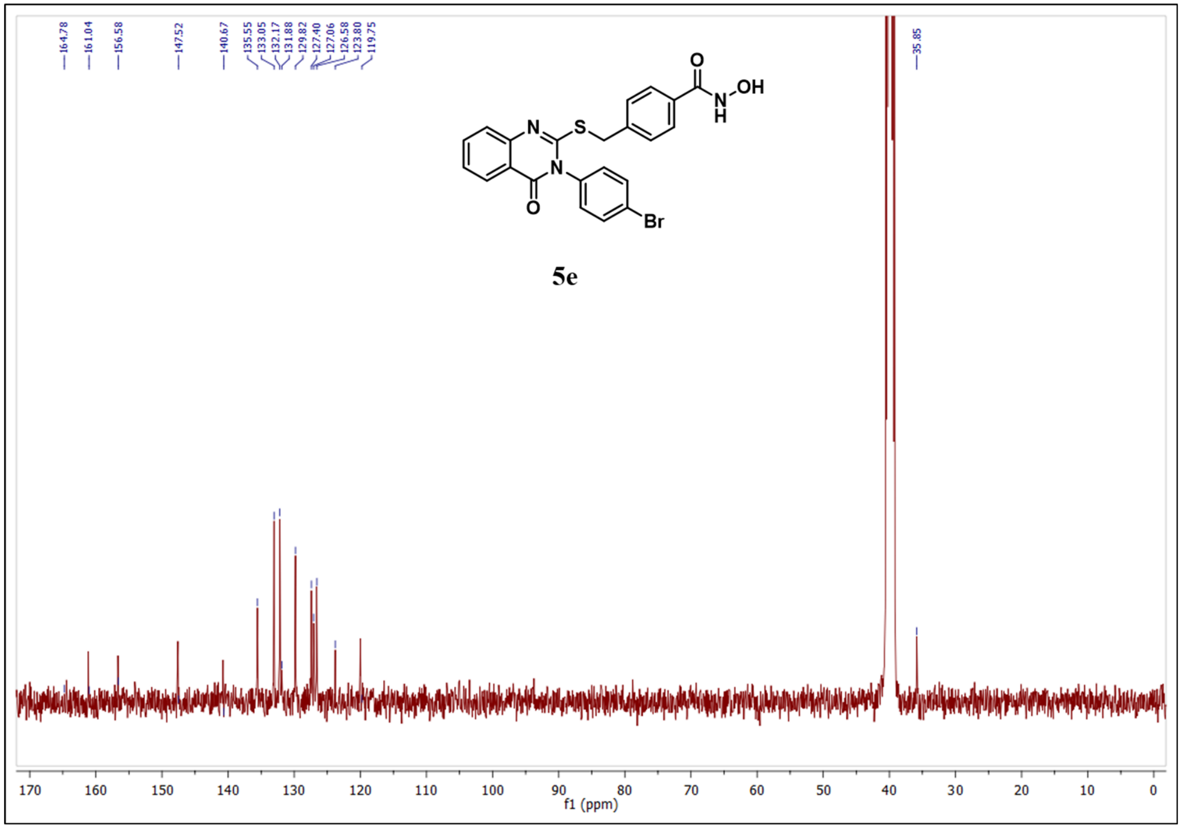

^{13}C NMR spectrum of compound **5e** (DMSO-*d₆* 101 MHz)

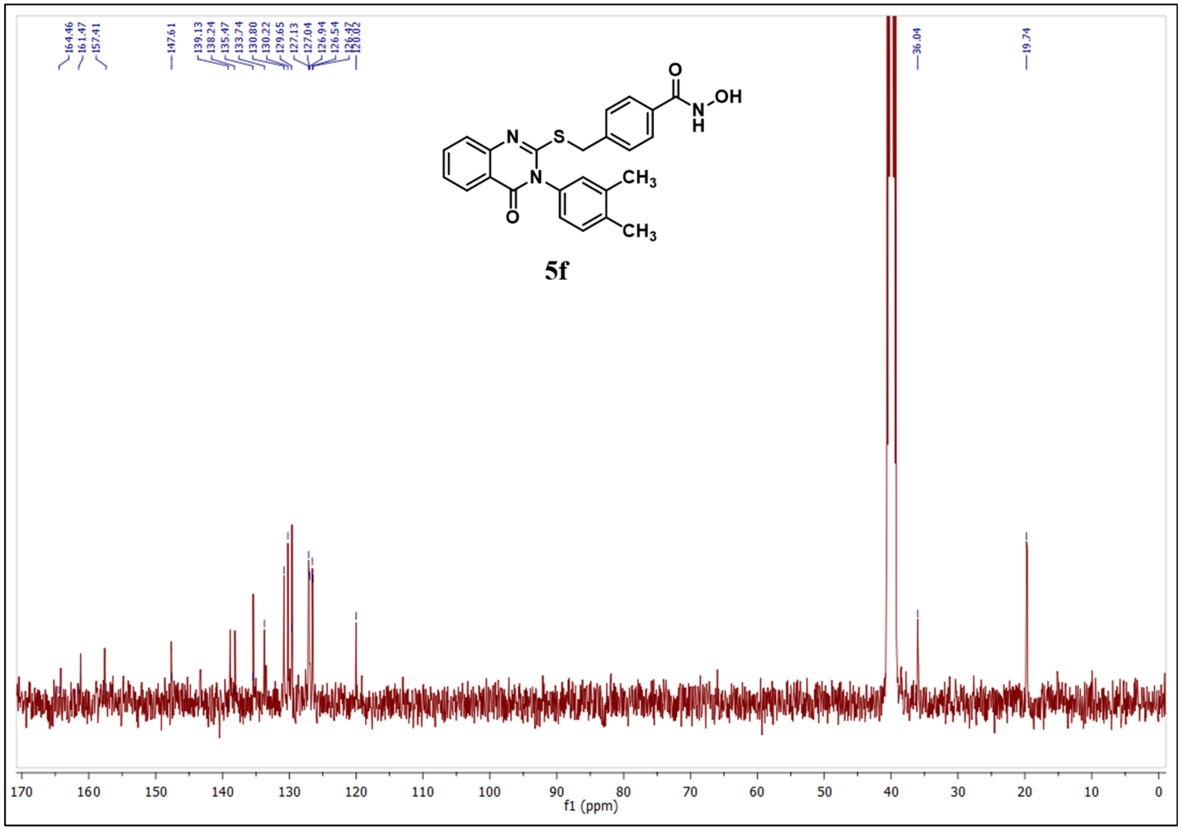

^{13}C NMR spectrum of compound **5f** (DMSO-d_6 101 MHz)
